# *Nmf9* Encodes a Highly Conserved Protein Important to Neurological Function in Mice and Flies

**DOI:** 10.1371/journal.pgen.1005344

**Published:** 2015-07-01

**Authors:** Shuxiao Zhang, Kevin D. Ross, Glen A. Seidner, Michael R. Gorman, Tiffany H. Poon, Xiaobo Wang, Elizabeth M. Keithley, Patricia N. Lee, Mark Q. Martindale, William J. Joiner, Bruce A. Hamilton

**Affiliations:** 1 Biomedical Sciences Graduate Program, University of California, San Diego School of Medicine, La Jolla, California, United States of America; 2 Department of Pharmacology, University of California, San Diego School of Medicine, La Jolla, California, United States of America; 3 Department of Psychology, University of California, San Diego School of Medicine, La Jolla, California, United States of America; 4 Center for Circadian Biology, University of California, San Diego School of Medicine, La Jolla, California, United States of America; 5 Division of Otolaryngology-Head and Neck Surgery, University of California, San Diego School of Medicine, La Jolla, California, United States of America; 6 Kewalo Marine Lab, University of Hawaii, Honolulu, Hawaii, United States of America; 7 Department of Cellular and Molecular Medicine, Department of Medicine, University of California, San Diego Moores Cancer Center, and Institute for Genomic Medicine, University of California, San Diego School of Medicine, La Jolla, California, United States of America; The University of North Carolina at Chapel Hill, UNITED STATES

## Abstract

Many protein-coding genes identified by genome sequencing remain without functional annotation or biological context. Here we define a novel protein-coding gene, *Nmf9*, based on a forward genetic screen for neurological function. ENU-induced and genome-edited null mutations in mice produce deficits in vestibular function, fear learning and circadian behavior, which correlated with *Nmf9* expression in inner ear, amygdala, and suprachiasmatic nuclei. Homologous genes from unicellular organisms and invertebrate animals predict interactions with small GTPases, but the corresponding domains are absent in mammalian *Nmf9*. Intriguingly, homozygotes for null mutations in the *Drosophila* homolog, *CG45058*, show profound locomotor defects and premature death, while heterozygotes show striking effects on sleep and activity phenotypes. These results link a novel gene orthology group to discrete neurological functions, and show conserved requirement across wide phylogenetic distance and domain level structural changes.

## Author Summary

Genome sequencing projects have identified large numbers of genes that encode proteins of unknown function. Many of these genes show strong evolutionary conservation, predicting important and well-conserved functions. A fraction of these show strong conservation of core domains but dynamic changes in other domains, predicting both conserved and lineage-dependent functions. Here we identify neurological functions associated with one such gene identified by a forward genetic screen in mice. We use recently developed genome editing tools both to confirm the mouse studies and to test comparative functions in a model insect, the fruit fly *Drosophila melanogaster*. Each of these species has a single homolog of this gene family, but differ by inclusion of a ras-association (RA) domain present in most invertebrate species but missing in mammals. Null mutations in both mice and flies produce neurological phenotypes, but while the mouse phenotypes are comparatively mild (vestibular deficits, mild tremor, hyperactivity, mild circadian phenotypes and abnormal fear learning–but normal viability and breeding), null flies rarely survive to adulthood and surviving flies have severe locomotor deficits. Interestingly, heterozygous flies have significant sleep-related phenotypes. Together, our results provide a detailed first look at comparative function for a gene lineage with an unusual evolutionary history.

## Introduction

The biological functions of many protein-coding genes remain unknown, often despite conservation across considerable evolutionary distance. Such “orphan” molecules may include genes whose functions affect fitness but not overt phenotypes in experimental settings, genes that affect overt but under-studied phenotypes, and/or genes that have been difficult to study due to unusual molecular properties, such as low level expression or poor physical annotation in genomes of well-studied organisms. Forward genetics offers one entry point into functional studies by highlighting those genes whose alterations produce measurable effects [1, 2]. A key challenge in de-orphanizing novel genes is to integrate their functions with observable sites of action, organism-level phenotypes and known cellular activities.

*Nmf9* and its homologs were essentially unknown prior to our studies. The *nmf9* mutation was recovered in a N-ethyl-N-nitrosourea (ENU) screen at the Neuroscience Mutagenesis Facility (NMF) of the Jackson laboratory [3, 4] based on tremor and vestibular phenotypes. Several exons of the gene we identify as *Nmf9* had been systematically annotated as *Ankfn1* (for ANK and FN3 domain containing), while other exons annotated as separate genes were identified only by clone names, based on non-overlapping partial cDNA clones from high-throughput screens [5, 6]. We selected *nmf9* for study as part of a long-standing interest in mice with unusual tremors and ataxias [7–11]. The unusual conservation pattern we identified among *Nmf9* homologs, along with recent innovations in genome editing [12–18], motivated additional studies to test conservation of function in mice and flies, including the generation of equivalent mutations at the same amino acid position in both species.

While our work was in progress, two groups reported identification of the *Drosophila* homolog in very different contexts. A transposon-based screen for low-sleeping mutants by Mark Wu and colleagues found insertions in a poorly conserved, variably included 5’ region of the gene, but concluded based on antibody studies that the resulting *wide-awake* (*wake*) alleles were null [19]. The same group also found that the fly gene interacts with and promotes cell surface localization of the fly GABA-A receptor encoded by *Resistance-to-dieldrin* (*Rdl*), through physical and genetic interactions. Independently, an RNAi screen for genes required for proper segregation of Numb during asymmetric cell division of sensory organ precursors carried out by Juergen Knoblich and colleagues identified the same gene, which they named *Banderuola* (*Bnd*). Intragenic deletion of most of the *Bnd* coding sequence resulted in pupal and early adult lethality [20]. This group also showed physical, genetic and functional interactions with *Discs-large 1* (*Dlg1*), a prototypical membrane-associated guanylate kinase (MAGUK). We will refer to the *Drosophila* gene by its current annotation symbol, *CG45058*, and to 5’ P element alleles as *wake*.

Here we use positional cloning, expression-guided behavioral studies, deep evolutionary constraint, and genome editing to define novel activities of *Nmf9* and its *Drosophila* homolog. Positional cloning of mouse *nmf9* identified a single point mutation at a splice donor sequence, resulting in exon skipping of a frame-shifting exon in *Nmf9*. The expression pattern of *Nmf9* in mouse brain suggested neural circuits that might be compromised in mutant animals. Behavioral tests confirmed deficits in vestibular function, fear learning, and circadian behavior. Female mice were more severely affected than males for several phenotypes. Sliding window analysis of relative constraint across the protein coding sequence showed that the skipped exon encodes the most evolutionarily constrained peptide sequence among Nmf9 homologs and genome editing of a single glycine to alanine at this site was sufficient to generate a non-complementing allele. Genome editing of the *Drosophila* homolog at three different sites produced null alleles that resulted in premature lethality and severe locomotor retardation in homozygotes. Remarkably, heterozygous flies showed mild activity and sleep-related phenotypes. Our studies thus show the first genetic, behavioral and molecular information for mammalian *Nmf9*, highlight functional importance of the most conserved sequence among its homologs, and resolve competing views regarding *CG45058* null phenotypes. These genetic, behavioral, and comparative studies provide a foundation for understanding activities of *Nmf9* homologues in broad context.

## Results

### Vestibular dysfunction in *nmf9* mice is sexually dimorphic and progressive

**Fig 1:**
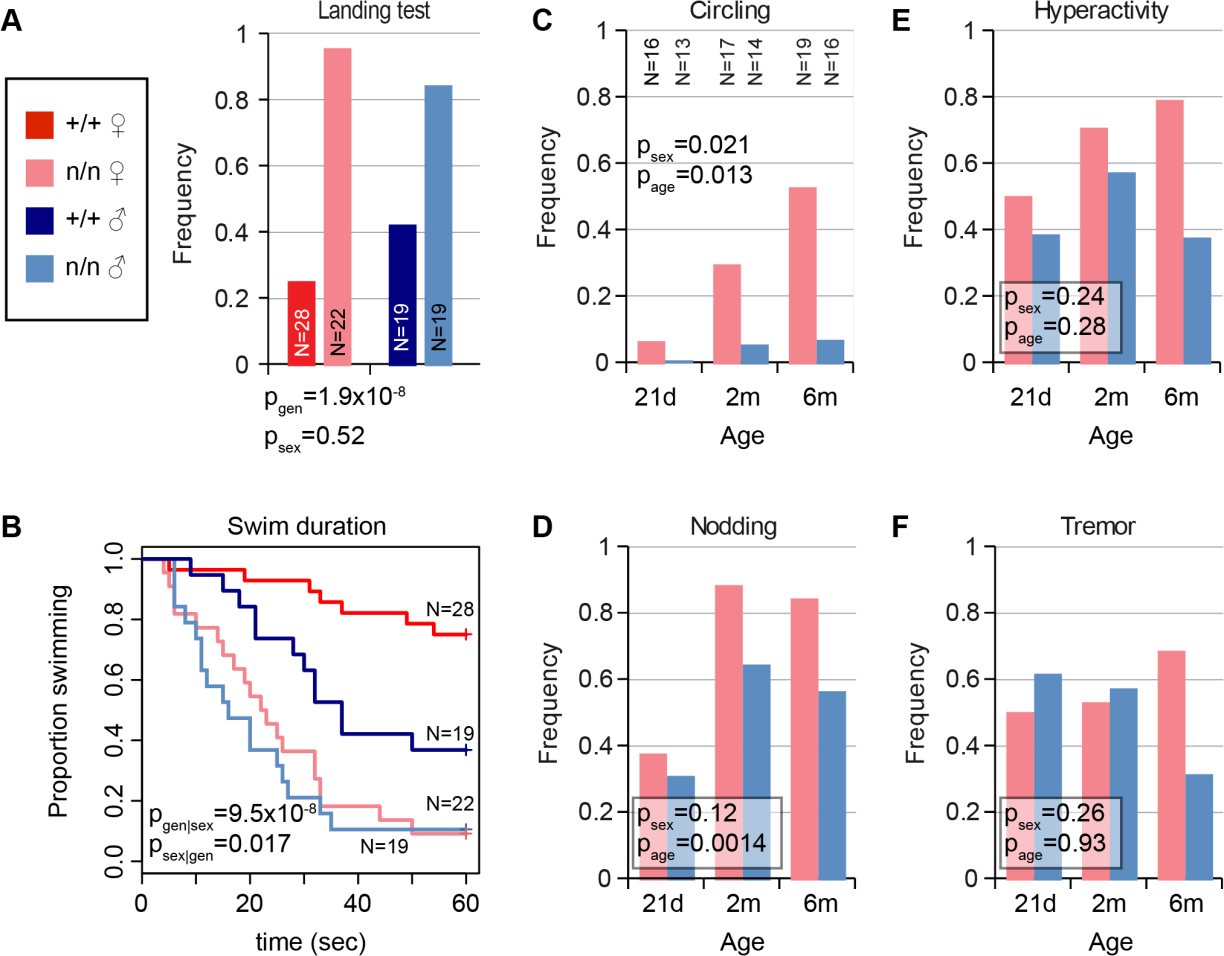
Mutant *nmf9* animals show age and sex-dependent vestibular dysfunction. (A) Landing test showed increased frequency of rotation or curling by *nmf9* homozygotes relative to control (+/+) littermates (Fisher's exact test, p = 1.9x10^-8^ between genotypes, 0.52 between sexes). (B) Forced swim test further showed vestibular dysfunction in *nmf9* relative to littermates (Asymptotic logrank test, p = 9.5x10^-8^ for geneotype stratified by sex) with a greater performance difference in females (p = 0.017 for sex stratified by genotype) due to lower performance among control males. Red, control; pink, mutant females. Navy, control; blue, mutant males. (C) Circling by mutant animals also showed significant sex bias (Fisher’s exact test, p = 0.021) and age-dependent progression (p = 0.013) in frequency. Sample sizes apply to panels C-F. (D) Head nodding also supported progression (p = 0.0014), but not significant sex bias (p = 0.12). Although (E) hyperactivity and (F) tremor were pronounced in mutant animals, effects of sex and age on did not reach statistical significance.

The *nmf9* mutation was recognized in a chemical mutagenesis screen based on tremor and vestibular phenotypes. In our evaluation, vestibular signs included circling, nodding, and head tilt (S1 Video) and abnormal landing and forced swim tests. Not every mutant was abnormal in every test, but all mutant animals were abnormal in at least one test and by visual inspection relative to co-isogenic non-mutant littermates. Visible head nodding, hyperactivity, and tremor were enhanced by light vertical acceleration or extended handling. In the landing test, animals were suspended by their tails and scored for trunk curling and attempts to rotate, rather than reaching for ground, by an investigator blinded to genotype. Mutant animals showed a significant increase in frequency of both trunk curling and rotation compared to littermate controls (Fig 1A). In the forced swim test, most control littermates swam with their snout above water for ≥1 min., while *nmf9* mutants typically were unable to remain righted above the surface and had to be rescued before 30 sec. to prevent drowning (Fig 1B). Differences between genotypes were more pronounced in females than males for vestibular phenotypes. The frequency and severity of circling and head nodding in mutant animals increased progressively from 21 days to 6 months (Fig 1C and 1D), though hyperactivity and tremor did not (Fig 1E and 1F); these phenotypes were essentially absent from control littermates. Histologically, however, mutant animals had grossly normal inner ear structures and did not show hearing impairment (S1 Fig), suggesting a mature functional, rather than a gross morphological, basis for vestibular defects.

### Positional cloning of *nmf9* reveals a novel gene expressed at low abundance

**Fig 2:**
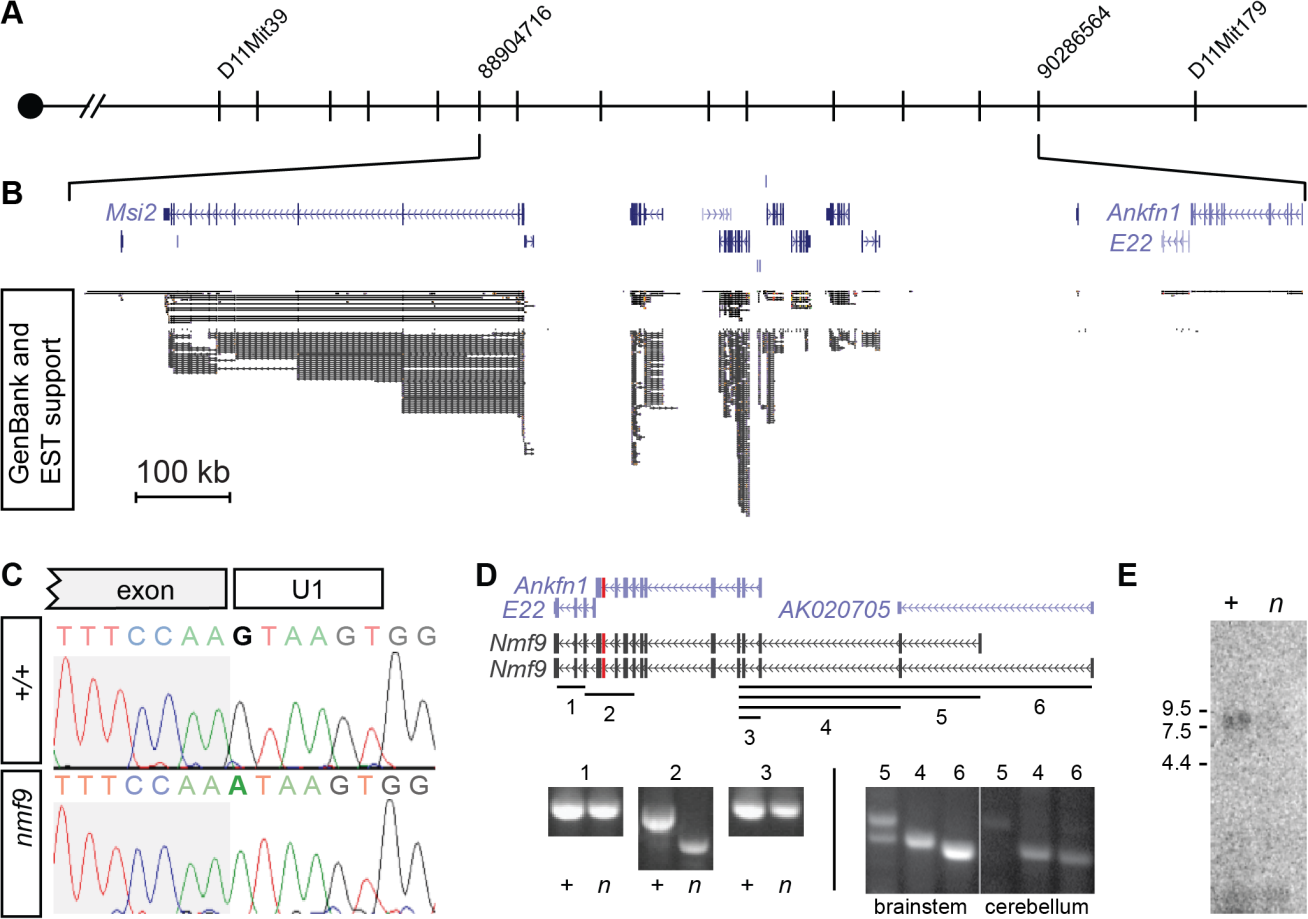
Positional cloning identifies *Nmf9*. (A) Recombination mapping places the *nmf9* mutation within a 1.3 Mb interval on Chr. 11. (B) Aligned UCSC Genome Browser window shows known and predicted genes in the non-recombining *nmf9* interval. Extent of EST support indicates poor sampling of putative genes *4932411E22Rik* and *Ankfn1*. (C) Sanger sequencing across the exon-intron junction shows a G-to-A transition in the essential GU splice donor adjacent to a frame-shifting exon in *Ankfn1*. (D) Schematic shows alignment of gene annotations from the UCSC genome browser (purple) with empirically determined transcripts (black) based on RT-PCR assays (numbered lines). Amplification and sequence analysis of assay 2 showed both inclusion of *Ankfn1* and *E22* in a single transcript in littermate control samples (+) and selective skipping of the mutated exon (red) in mutant samples (*n*). Additional assays indicated based on conservation, predicted exons and 5’ RACE showed variable inclusion of exons 5’ to the *Ankfn1* annotation. (E) Northern blot shows a single major band for *Nmf9* poly(A)^+^ RNA from brain ~9 kb, with reduced level in *nmf9* mutant (*n*) compared to control (+) littermate brain RNA.

We mapped the *nmf9* mutation by recombination in ~1000 F2 progeny from crosses between B6-*nmf9* and four mapping strains (Fig 2A). Intercross progeny included 332 from AKR/J, 245 from BALB/cJ, 371 from C3H/HeJ, and 119 from DBA/2J that were typed by PCR ([21] and S1 Table). The data confirmed a fully penetrant, recessive phenotype with no evidence of segregating modifier genes. Exclusion mapping placed *nmf9* within a 1.3 Mb interval (Chr11: 88,240,426–89,538,743 in GRCm38/mm10 assembly) that included several well-annotated genes. We previously estimated the frequency of induced nucleotide mutations in the NMF screen as ~1/Mb [4], which predicts a very low probability of confounding functional mutations in an interval of this size [22]. Sanger sequencing of all canonical and EST exons (Fig 2B) in mutant and littermate control identified a single mutation: a G-to-A transition in the splice donor U1 binding site of a frame-shifting exon of predicted gene *Ankfn1* (Fig 2C). RT-PCR, homologous cDNAs from other species, and detailed in situ hybridization patterns indicated that the major transcript of this gene comprised both *Ankfn1* and *4932411E22Rik* annotations as well as additional 5’ exons (Figs 2D and S2). We refer to this transcript and locus as *Nmf9* to avoid confusion with the narrower definition of *Ankfn1*. RT-PCR across EST and predicted exons showed low overall abundance (requiring polyA^+^ RNA for detection from whole brain), skipping of the frame-shifting exon adjacent to the mutated U1 site, and variable utilization of alternative 5’ ends (Fig 2D). RNA gel blot hybridization showed a single major size form and confirmed that while the transcript had very low abundance in control brain, the mutant transcript had still lower levels in *nmf9* homozygote brains, consistent with predicted nonsense-mediated decay for the frame-shifted splice product (Fig 2E). While it remains possible that other transcripts exist at low levels, our data support a single major open reading frame, with some variation at the 5’ end.

### *Nmf9* is expressed in discrete sensory and CNS structures

**Fig 3:**
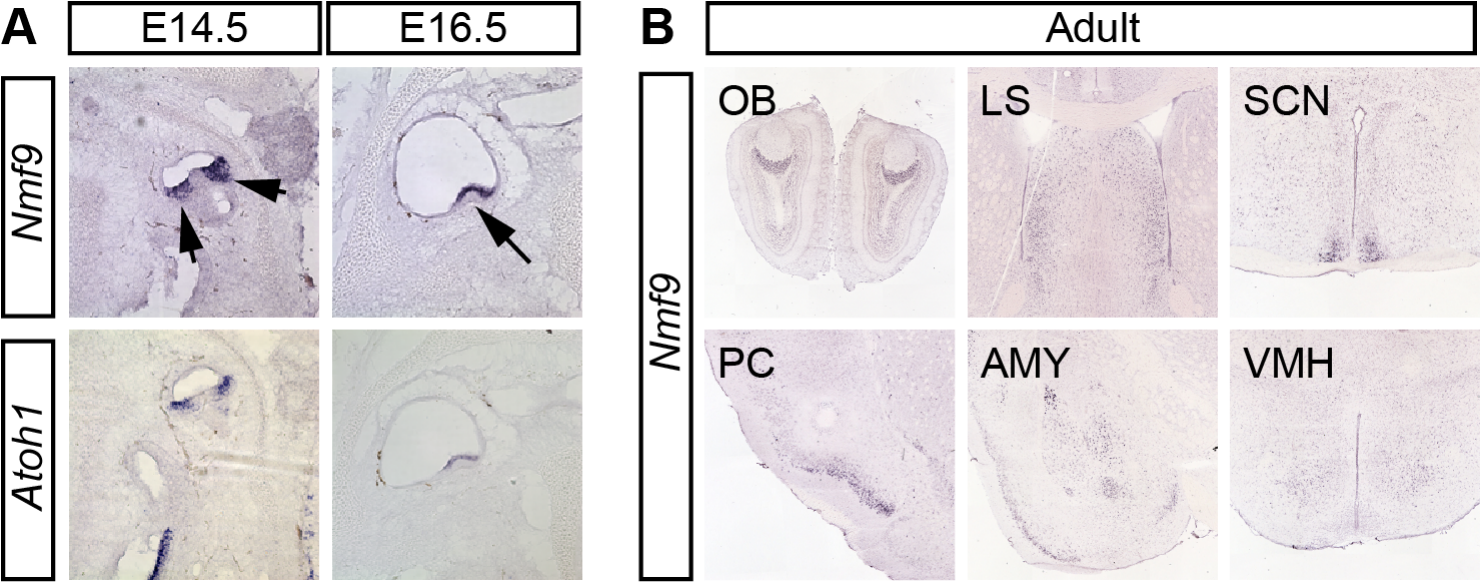
*Nmf9* expression pattern predicts new sites of function. (A) *In situ* hybridization to adjacent sections in E14.5 and E16.5 embryos show that *Nmf9* and *Atoh1*, a marker of hair cell progenitor were expressed in the same population of cells (indicated with arrows). Scale bar = 500 μm. (B) Panels from the Allen Brain Atlas [25] show *Nmf9* RNA enriched in distinct structures of adult brain, including accessory olfactory bulb (OB), piriform cortex (PC), lateral septum (LS), amygdala (AMY), suprachiasmatic nucleus (SCN) and Ventromedial hypothalamus (VMH).

To define potential sites of *Nmf9* action, we examined its pattern of expression. In situ hybridization to both male and female embryos and RT-PCR from dissected tissue showed *Nmf9* expression largely restricted to the nervous system. In vestibular inner ear, *Nmf9* expression fully overlapped *Atoh1* (Fig 3A), a well-studied marker for hair-cell progenitors [23, 24]. In situ hybridization showed that *Nmf9* was expressed as early as E14.5 in inner ear, nasal epithelium, ventricular zone, and the spinal cord (S2 Fig). In adult brain, both our data and data from the Allen Brain Atlas [25] showed expression enriched in a few centers (Fig 3B), including the accessory olfactory bulb (OB), piriform cortex (PC), lateral septum (LS), amygdala (AMY), suprachiasmatic nucleus (SCN), and modest enrichment in ventral medial hypothalamus (VMH). Probes corresponding to *Ankfn1* and *4932411E22Rik* annotated exons showed the same detailed pattern in our data (S2 Fig) and in comparable data from the Allen Brain Atlas, further supporting a single transcription unit. Identification of *Nmf9* pattern in mouse brain allows behavioral tests of neurological function for each of the major sites of expression, providing a structured approach to defining additional phenotypes in *nmf9* mice.

### Fear learning and circadian behaviors are impaired in *nmf9* mice

To probe the functional significance of *Nmf9* expression in neuroanatomical structures outside of the vestibular system, we tested *nmf9* and littermate (+/+) control mice on selected behavioral tasks. Each task included enough male and female animals to assess potential sex differences, as female mice had shown earlier and stronger vestibular defects than males. A summary of statistical analyses is presented in S2 Table.

#### AMY

**Fig 4:**
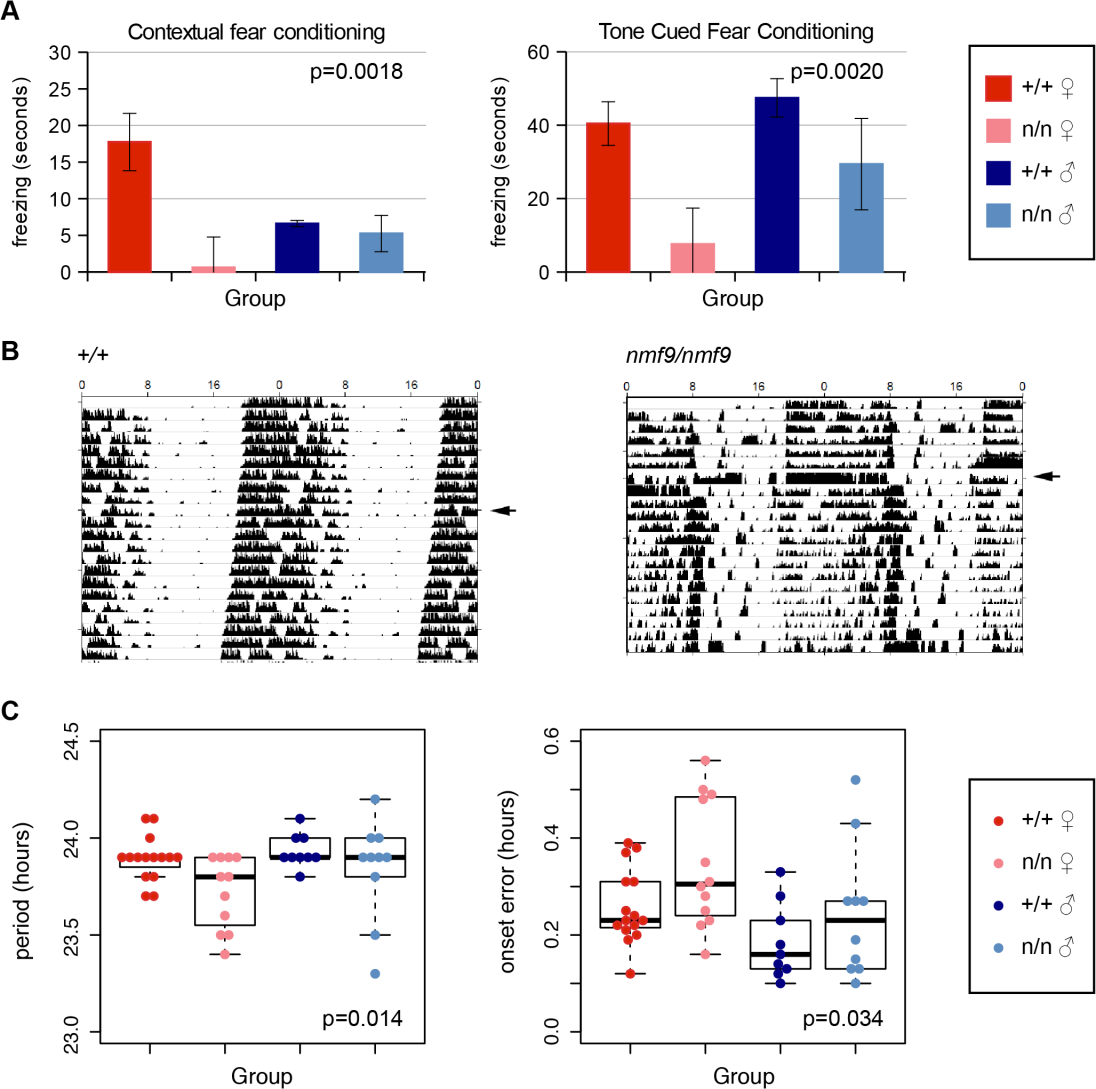
*Nmf9* is important to fear learning and circadian rhythm. (A) Mutant animals showed decreased freezing behavior in both tone cued and contextual fear conditioning. (2-factor ANOVA p = 0.0018 for genotypes in contextual conditioning, 0.002 in tone-cued conditioning. Sex effects were non-significant, p = 0.29 in contextual, p = 0.09 in tone-cued, N = 14 +/+ female, 8 mutant female, 11 +/+ male, 8 mutant male, although the interaction between genotype and sex was supported for contextual fear conditioning, p = 0.026.) (B) Double-plotted actograms of running-wheel counts of mutant and wildtype from the same litter housed in 12 hour light: 12 hour dark (LD) cycle and continuous dark (DD) illustrates less robust circadian rhythm in a more severely affected animal. Arrowheads indicate transfer to DD. (C) Mutants showed a small but significant decrease in period length (p = 0.014) and an increase in period onset error (p = 0.034) during DD (2-factor ANOVA; effects of sex p = 0.14 for period length, p = 0.012 for onset error, with larger deviation in females. N = 14 wildtype females, 12 mutant females, 9 wildtype males, 10 mutant males).

The amygdala is essential for fear learning. In both contextual and cued fear conditioning paradigms, *nmf9* mutants showed diminished freezing behavior after training compared to littermates, indicating decreased fear learning (Fig 4A). Similar to vestibular defects, fear learning was more strongly affected in females than in males.

#### SCN

The suprachiasmatic nuclei are essential for classic circadian behaviors, in which *nmf9* mutant animals showed significant defects. Animals were monitored for activity on a wheel running apparatus in 12 h light: 12 h dark (LD) and in constant dark (DD) conditions. The most severely affected *nmf9* mice showed less robust consolidation of activity into nighttime hours when compared to +/+ littermates (Fig 4B). Mutants in aggregate showed both a slight shortening of period and an increase in onset error in DD (Fig 4C). Hyperactivity did not significantly affect activity counts in either LD or DD housing conditions (Fig 4B and S3 Table).

#### LS

**Fig 5:**
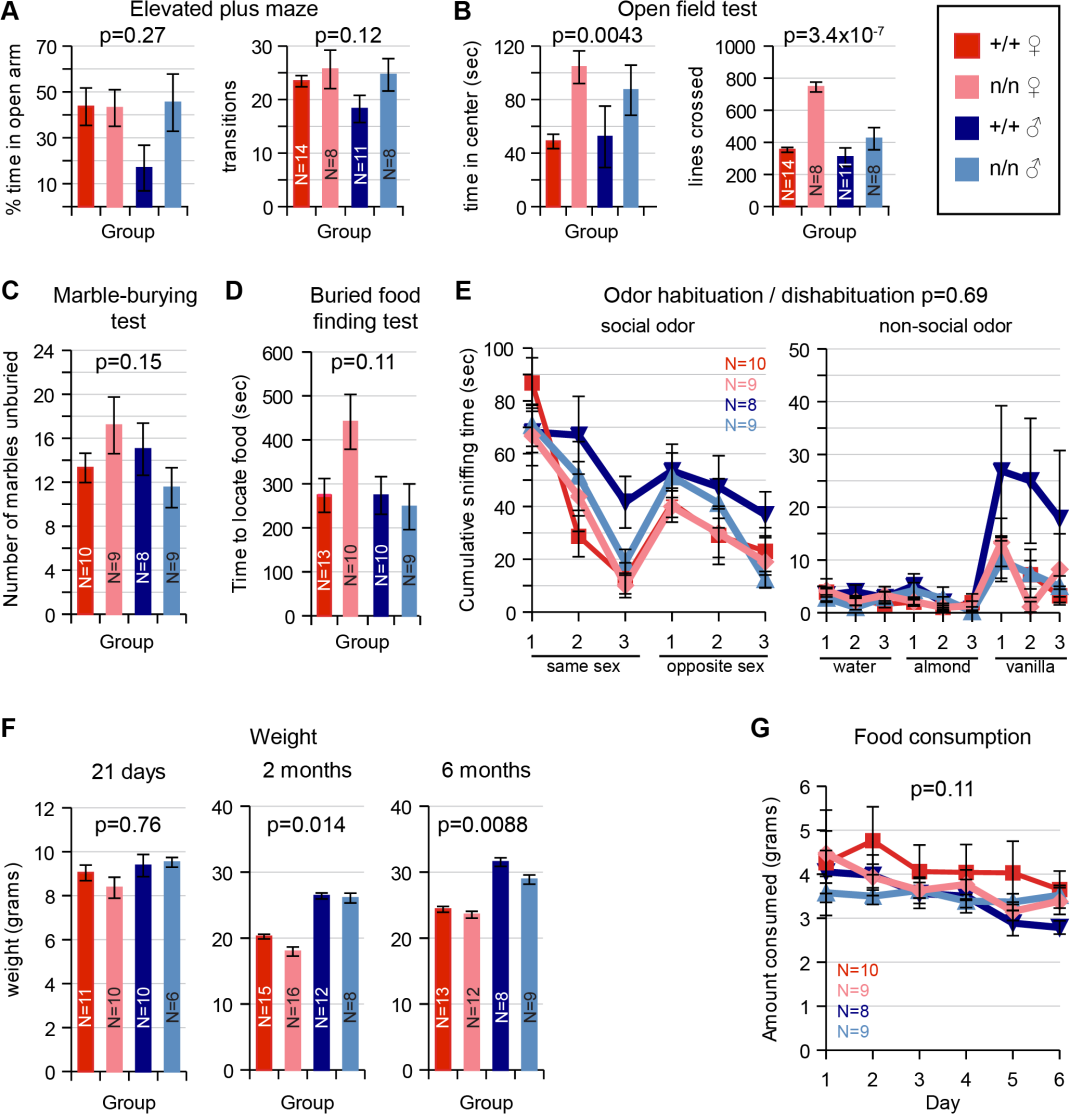
Tests of LS, PC/olfactory pathway, and VMH. Behaviors that depend on *Nmf9*-expressing circuits were assayed and assessed by 2-factor ANOVA or MANOVA for sex and genotype. Number tested for each group (N) is indicated in each bar or a legend to line graphs. (A) Elevated Plus Maze behaviors were not significantly different between control (+/+) and mutant (n/n) littermates (Percent time in open arm p = 0.27 for genotype, p = 0.22 for sex; Number of transitions p = 0.12 for genotype, p = 0.23 for sex). (B) In the Open Field Test, mutant animals showed increased time spent in center, but potentially confounded by increased number of line crosses due to hyperactivity (Time in center p = 0.0043 for genotype, p = 0.82 for sex; number of transitions, 3.4x10^-7^ for genotype, p = 0.0004 for sex). (C) No significant difference between genotypes was detected for the Marble Burying Test (p = 0.15 and 0.68 for genotype and sex, respectively). (D) While mutant females took somewhat longer in olfactory-dependent Buried Food Finding Test, this was did not reach conventional significance (p = 0.11 for genotype, 0.09 for sex). (E) Similarly, Odor Habituation / Dishabituation Tests did not support a significant difference (MANOVA, p = 0.69 for genotype, p = 0.30 for sex). (F) Weight was significantly different by genotype at 2 months (ANOVA, p = 0.014) and remained significant at 6 months (p = 0.0088), including well-known sex differences, while food consumption (G) was not significantly different (p = 0.11 for genotype, 0.75 for sex).

Measures of innate anxiety, which are dependent on the lateral septum, appeared intact in *nmf9* mutants. Behavior on an elevated plus maze detected no significant difference between genotypes (Fig 5A). In an open field test, *nmf9* mutants spent more time in the center of the open field, which could indicate a functional change in LS, but the number of line crossings was also significantly higher, suggesting that the hyperactivity and circling behaviors in *nmf9* confound a simple interpretation of the open field results (Fig 5B). Marble burying, a third test of anxiety-related behavior that is less affected by activity, showed no significant difference between genotypes (Fig 5C).

#### PC, OB and olfactory epithelium

Piriform cortex plays an important role in olfactory sensing and behavior. Although PC, OB and olfactory epithelium showed significant *Nmf9* expression, mutants showed no significant difference from littermates in behavioral tests of olfactory function, which included a buried food finding task (Fig 5D) and odor habituation / dishabituation tests with either social or non-social odors (Fig 5E).

#### VMH

Ventromedial nuclei of the hypothalamus are important to satiety and feeding behavior, among other functions. Mutant animals show a slight but significant decrease in weight compared to littermates at 2–6 months (Fig 5F) but no significant difference in the amount of food consumed (Fig 5G). While these results were also potentially confounded by hyperactivity and energy expenditure, the small effect sizes suggested limited, if any, impact on feeding behavior.

Taken together, results of these behavioral data support selective functional impairments in anatomical circuits that express *Nmf9*.

### Pattern of conservation among *Nmf9* homologs indicates modular architecture and novel domains

**Fig 6:**
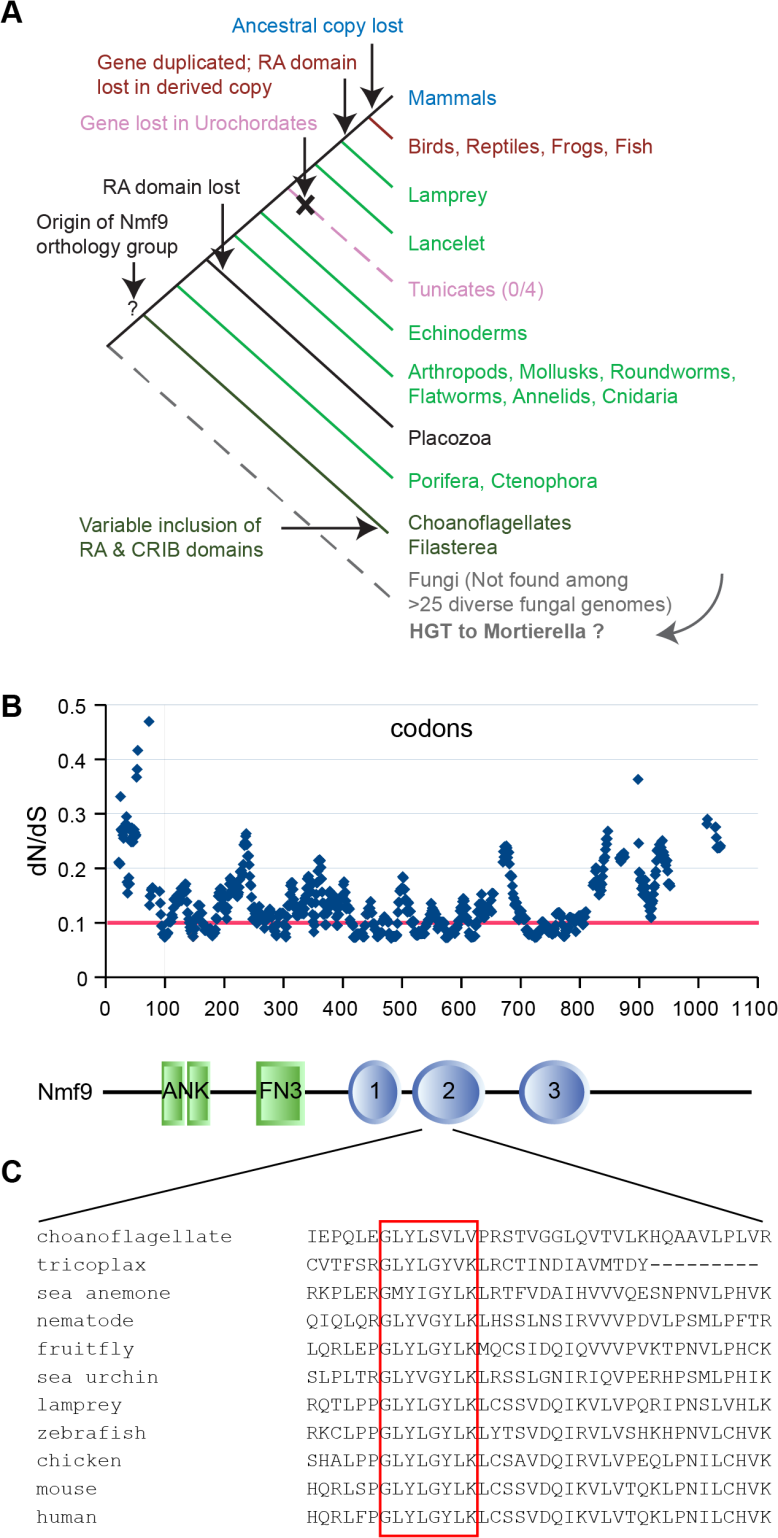
Nmf9 protein is highly conserved in Metazoans. (A) Nmf9 homologs exist for all branches of Metazoa (except Urochordates) and several non-animal Holozoa. A single example in fungi (*Mortierella verticillata*) may represent horizontal gene transfer. (B) Sliding window analysis of 14 approximately full-length metazoan homologs highlights known motifs and predicts three novel domains where the most highly conserved individual residues tend to cluster. Threshold line (red) indicates 25% percentile of values. Aligned schematic shows motif annotations (rectangles) and constrained intervals (ovals) in relation to the sliding window plot. (C) The most highly conserved amino acid sequence (red box) was in domain 2 and encoded by the exon skipped in *nmf9* mRNA.

Homologs of *Nmf9* across wide taxonomic boundaries showed strong patterns of conservation that highlight select domains, as well as lineage-restricted modulation of domain architecture (Figs 6A and S4). Homologs were identified by reciprocal BLAST/BLAT searches in sequenced genomes of nearly all metazoan lineages, including placozoa and porifera, and in at least some choanoflagellates and filasterea, sister groups to animals that diverged from the lineage leading to animals after the split between animals and fungi. Choanoflagellate (*M*. *brevicollis*, *S*. *rosetta*) and filasterea (*C*. *owczarzaki*) homologs included an N-terminal CRIB domain (associated with binding to Cdc42/Rac subfamily small GTPases), a C-terminal Ras-association (RA) domain, or neither. A single instance in fungi–comprising only a choanoflagellate-like copy of the conserved, non-motif domains–was found in *Mortierella verticillata*, but not in basal or sister lineages, and might therefore represent a horizontal gene transfer event rather than an earlier origin of the gene genealogy. Sequenced invertebrate animal genomes had a single *Nmf9* homolog, except for an apparent loss in the urochordate lineage (0/4 species). Invertebrate homologs included an RA domain, except for the single placozoa sequence and a few genomes with incomplete assembly, but none included a CRIB domain. Jawed vertebrates basal to mammals contained two homologs: an ancestral copy with the RA domain and a derived copy without it. Mammalian genomes had only the derived copy. Most animal homologs occur in poorly annotated regions of their respective genome assemblies, limiting the number of complete sequences available among homologs we examined.

Analysis of evolutionary constraint among 14 animal species spaced by known evolutionary distance [26] confirmed conservation of the annotated ankyrin and fibronectin type 3 motifs, but also predicts three additional regions of unknown biological function under equal or stronger constraint (Fig 6B). Novel domain 2 was the most conserved region in the entire protein, including a GLYLGYLK sequence that is nearly invariant among animals and whose first glycine was the most highly conserved residue (Fig 6C). This region contained no motif annotation in current databases nor predicted post-translational modification sites. In a detailed analysis of 113 homologous sequences, Domain 2 was the most highly conserved sequence both among ancestral homologs and among derived homologs, with the GLYLGYLK signature providing the strongest sliding window support in both (S5 Fig).

In situ hybridization studies in diverse animal species suggested the potential for a conserved neuronal function. In contrast to mouse, the homolog in *D*. *melanogaster* appeared more broadly expressed during development, including both CNS and tissues outside of the nervous system (S6 Fig). Expression in the sea anemone *N*. *vectensis* also showed neural expression: in the planula, highest expression was in the apical tuft, a larval chemosensory organ, and in the pharyngeal nerve ring; in the polyp stage highest expression was in the endoderm, the predominant region of the net-like adult nervous system [27]. Ancestral and derived homologs may also differ in expression pattern. In the model fish *D*. *rerio*, the ancestral homolog appeared broadly expressed, as in *D*. *melanogaster*, while the derived homolog appeared more strictly localized, including distinct expression in the developing inner ear.

### Genome-edited alleles confirm functional importance of conserved domain 2

**Fig 7:**
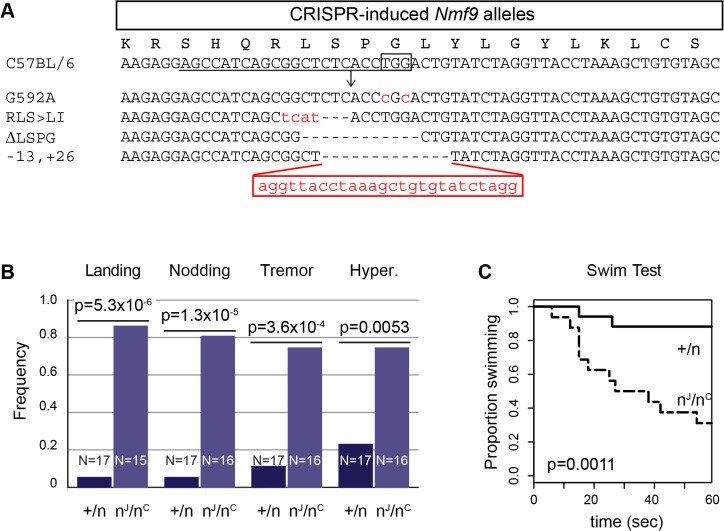
Genome edited alleles show importance of conserved domain 2 and confirm identity of *nmf9*. (A) CRISPR/Cas9 target sequence is underlined, PAM site boxed, and predicted cut site shown as an arrow. Sequences of four induced alleles selected for further analysis are shown; deleted bases are indicated by dashes and insertions/substitutions by red lower-case letters. The first glycine residue, targeted for replacement, was at position 592 relative to RefSeq protein NP_001074402. (B) Results of the landing test with induced mutations heterozygous either to a coisogenic nonmutant allele (+/n) or to the original *nmf9* Jackson allele (n^J^/n^C^) showed non-complementation (p = 5.3x10^-6^, Fisher’s exact test). Similarly, head nodding (p = 1.3x10^-5^), tremor (3.6x10^-4^), and hyperactivity (p = 0.0053) were more frequently observed in trans-heterozygotes for *nmf9* and CRISPR-induced alleles than in controls. (C) Time-to-event in the forced swim tests also showed non-complementation between edited and the original *nmf9* alleles (Asymptotic logrank test, p = 0.0011).

To confirm the gene identification of *Nmf9* and to test the functional importance of conserved domain 2, we used CRISPR/Cas9-mediated genome editing in mouse one-cell embryos [17, 28] to target mutations to the conserved GLYLGYLK region. We recovered 26 G0 animals, of which 24 survived to adulthood. Sequencing of a 550-bp PCR product encompassing the target site showed that 21 G0 animals were edited on both alleles, 4 were edited on only one allele, and one was not determined. Among 21 edited on both alleles, 17 appeared to be homozygous (or possibly heterozygous to an allele that precluded amplification, such as a large deletion). G0 mice with both alleles edited to frameshift or other clearly deleterious lesions phenocopied the overt vestibular phenotypes of *nmf9* (Table 1). Four predicted pathogenic mutations were used for complementation tests with the original *nmf9* allele (Fig 7A and Table 2). In aggregate, G1 and later progeny carrying one edited allele heterozygous to the original *nmf9* mutation failed to complement in both the landing test and forced swim test (Fig 7B and 7C), confirming correct gene identification of *nmf9*. The first glycine of the GLYLGYLK sequence was conserved through metazoa and holozoan sister groups. Strikingly, an alanine substitution at this residue failed to complement *nmf9*. Although homozygotes for this allele did not show a strong phenotype, G-to-A/*nmf9* heterozygotes had the tremor, hyperactivity, and vestibular dysfunction characteristic of *nmf9* homozygotes (Table 2 and S2 Video). These results support the functional importance of the highly conserved domain at a neurological level, even under the relaxed constraints of inbred laboratory mice.

**Table 1:** Mouse *nmf9* alleles generated by CRISPR/Cas9-mediated genome editing.

Mouse	Sex	Apparent Zygosity	Genotype	Mutations	Hyper	Nod	Tremor	Landing	Fig 7 label
1 (Dead)	-	Homozygous	-6	CTCTCAC>A	-	-	-	-	
**2**	F	Homozygous	+13	-CTCACCTGGACTGTATCT + 31	2	2	0	1	-13,+26
3	M	Homozygous	substitution	C>A	0	0	0	0	
**4**	M	Homozygous	substitution	TGG>CGC	0	0	0	0	G592A
5	F	Heterozygous	not determined	not determined	0	0	0	0	
6	F	Heterozygous	-33	-TCTCACCTGGACTGTATCTAGGTTACCTAAAGC	2	2	0	2	
7	F	Homozygous	-7	GGCTCTCA>T	1	1	1	0	
8	M	Homozygous	+24	+TGACTGCTTTAGGTAACCTAGATA	2	1	1	2	
9	M	Homozygous	-10	-GCTCTCACCT	0	1	1	2	
10	M	Trans-Heterozygous	+1/-4	+C/-CTCT	2	2	2	2	
11	F	Homozygous	>300bp insertion	[not depicted]	1	2	0	2	
12	F	not determined	not determined	not determined	0	1	2	2	
**13**	F	Homozygous	-12	-CTCACCTGGACT	2	2	1	2	-∆LSPG
14	F	Homozygous	-4 & substitution	-ACCT and 2 substition	2	2	1	2	
15	F	Homozygous	+98 & substitution	+98 & TGG>CGC	2	2	1	2	
16	M	Heterozygous	-3	CTCTCACCTGG>ATACCCGC	1	0	0	0	
17	M	Homozygous	+1	+T	1	2	0	2	
18 (Dead)	F	Heterozygous	-12	-TCAGCGGCTCTC	-	-	-	-	
19	F	Homozygous	-220 & substitution	TGG>CGC & -220	1	0	0	0	
20	F	Homozygous	-169	[not depicted]	2	1	0	2	
21	F	Heterozygous	-6	CTCTCACCT>ACA	2	2	1	2	
22	F	Homozygous	-4	-TCTC	2	2	2	1	
23	F	Homozygous	inversion	[not depicted]	0	2	1	2	
**24**	F	Trans-Heterozygous	-11/complex	CCATCAGCGGCTCTCACCT>CTCTCCCT	2	1	1	1	
25	F	Trans-Heterozygous	+1/-4	+C/-CTCA	0	1	0	1	
**26**	F	Homozygous	-3	GGCTCTC>TCAT	0	0	0	2	RLS>LI

Phenotypes are noted as 2 for strong, 1 for mild, or 0 for not evident.

**Table 2:** Allele-level data and p-values for non-complementation of *nmf9* by CRISPR-induced mutations.

Genotype	Landing	Nodding	Tremor	Hyper.	Swim ≥60s	Avg. # Tests Failed
G592A/+	0/5	0/5	1/5	2/5	0/5	0.66
G592A/*nmf9*	4/5	4/5	4/5	4/5	3/5	3.80
RLS->LI/+	1/7	1/7	1/7	1/7	2/7	0.86
RLS->LI/*nmf9*	1/1	0/2	0/2	0/2	1/2	1.00
∆LSPG/+	0/3	0/3	0/3	1/3	0/3	0.33
∆LSPG/*nmf9*	3/4	4/4	3/4	4/4	3/4	4.25
-13,+26/+	0/2	0/2	0/2	0/2	0/2	0.00
-13,+26/*nmf9*	5/5	5/5	5/5	4/5	5/5	4.60
SUM mut/+	1/17	1/17	2/17	4/17	2/17	0.83
SUM mut/*nmf9*	13/15	13/16	12/16	12/16	11/16	3.74
Fisher exact p-value	5.3x10^-6^	1.3x10^-5^	3.6x10^-4^	0.0053	0.0013	

Frequency with which animals of the indicated genotype were called abnormal by a trained observer blind to genotype is indicated for each test and allele combination, as is the average number of the five tests for which a given genotype was considered abnormal. P-values are for the one-sided Fisher’s Exact Test of the null hypothesis that mutant/*nmf9* heterozygotes are not more often affected than mutant/+ heterozygotes, based on sums across all five induced alleles.

### Null alleles of the *Drosophila* homolog have viability and locomotor deficits and heterozygous effects on sleep

**Fig 8:**
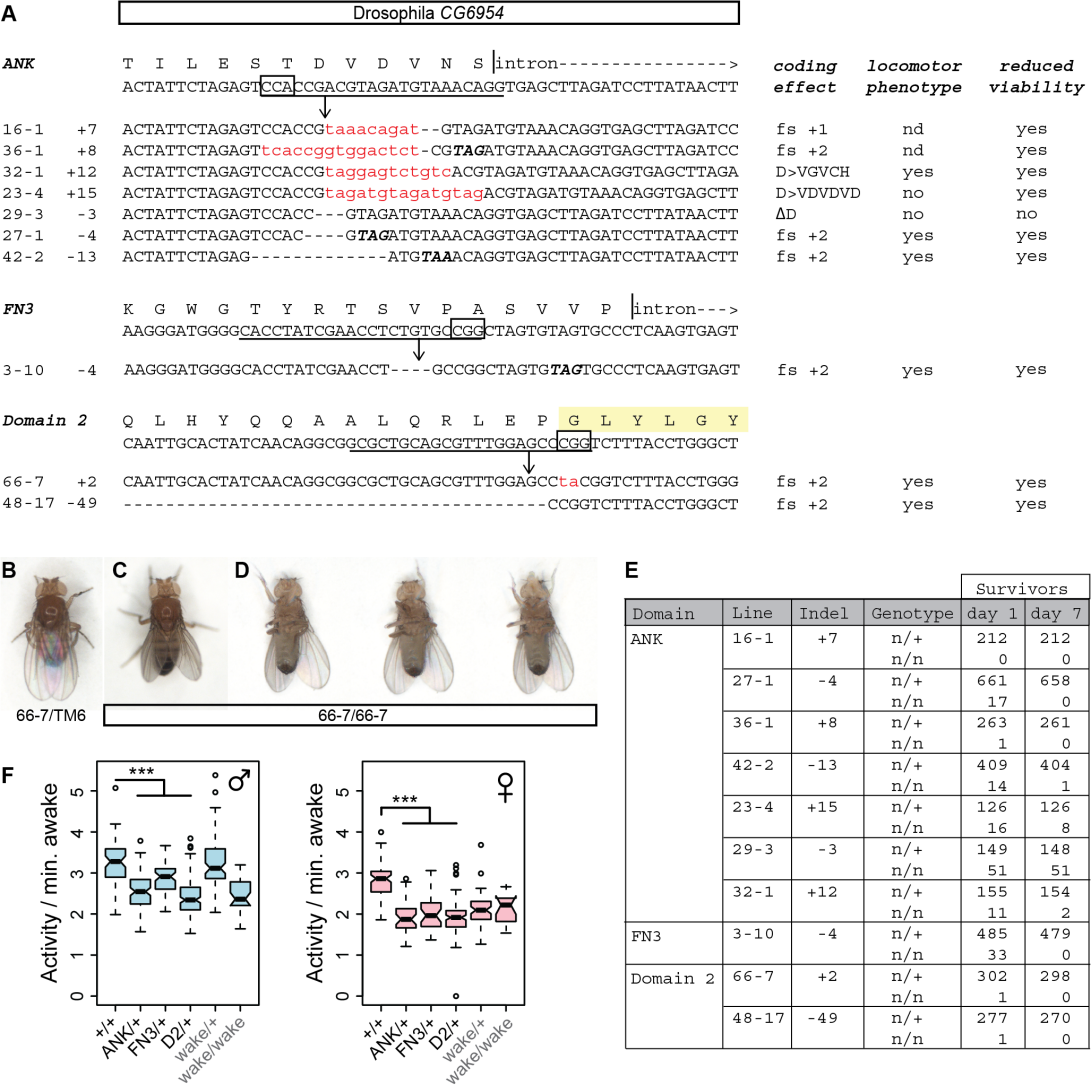
Genome editing of *Drosophila CG45058* exons results in severe locomotor deficits and early death. (A) Target sites and recovered alleles are shown for separately targeted exons encoding the first ANK repeat, FN3 domain, and the GLYLGYLK peptide. (B) Posture of a heterozygous control fly. (C) Posture of a mutant fly homozygous for a frame-shifting mutation in conserved domain 2. Note wings held up at an angle with respect to the body. (D) The same mutant fly subsequently fell over and moved legs in an uncoordinated fashion. (E) Survival frequencies at eclosion and 7 days post-eclosion. (F) Total waking activity (beam breaks per minutes awake) for males (blue) and females (pink) of the indicated genotypes. Heterozygotes for framshifting mutations in *CG45058* ANK, FN3 or conserved domain 2 consistently showed reduced activity relative to +/+ control, similar in magnitude to *wake* (EY02219) homozygotes. *** p<2.2x10^-16^.

To test conservation of function at an organismal level, we similarly edited the *Drosophila* homolog of *Nmf9*, *CG45058*, at conserved sites in three different exons: the first ANK repeat, the FN3 domain, and conserved domain 2 (Fig 8A). Apparent null and other predicted deleterious mutations induced at each site showed a consistent, severe adult locomotor phenotype and reduced viability. Newly eclosed homozygotes were predominantly stationary and most could be placed unanesthetized on a cardboard surface without the animal attempting to fly or walk (Fig 8B, 8C and 8D). Most held their wings at unusual postures, either up or slightly down from the horizontal plane. When these animals did move, they typically fell over onto their backs and kicked their legs without apparent coordination. Mutant adults had very poor survival, even in uncrowded, horizontal vials (Fig 8E). In addition to three independent sets of alleles with consistent phenotype, deficiency mapping with two distinct alleles induced at conserved domain 2 confirmed that the severe locomotor defects map to the *CG45058* locus as a loss of function (S7 Fig). These data conflict with an interpretation that *wake* alleles of this locus, which do not have strong locomotor phenotypes, are null [19]. In contrast, these data are fully consistent with the *bnd* intragenic deletion allele as null [20].

**Fig 9:**
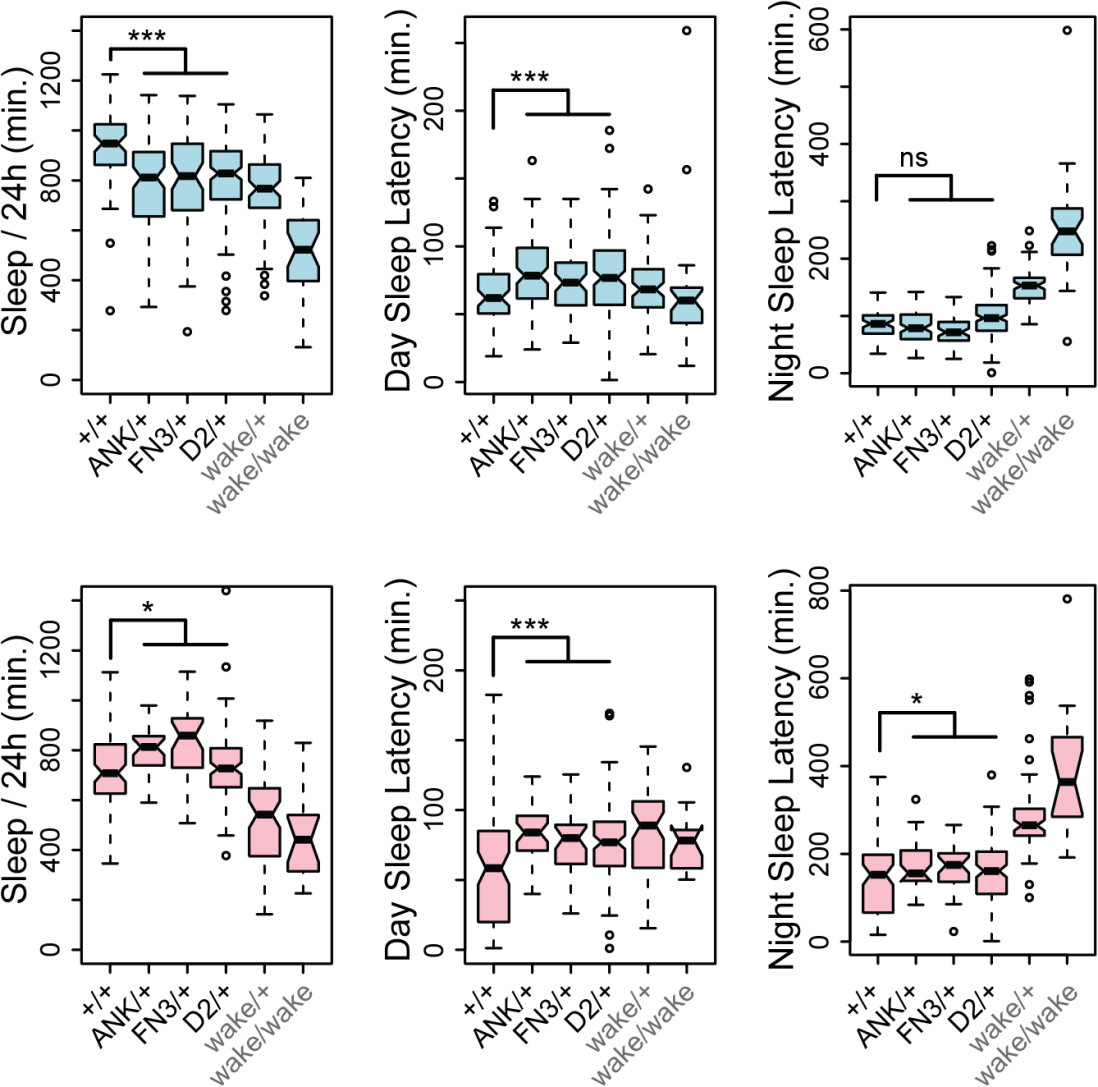
*CG45058* null heterozygotes have abnormal sleep patterns. Male heterozygotes (blue graphs) recorded less total sleep per 24 hr. period and slightly longer latency to first sleep bout after lights on (daytime sleep latency) than +/+ controls, but no significant difference in latency after lights off (nighttime sleep latency). By contrast female heterozygotes (pink graphs) recorded slightly more overall sleep than +/+ controls and slightly longer daytime and nighttime sleep latencies. Heterozygous *wake* P-element alleles (EY02219, KG02188, KG08407 and MI02905 insertion lines) and a homozygous *wake* allele (EY02219) were recorded as a comparison. * p<0.025, *** p<5x10^-4^, ns p = 0.78, Wilcoxon rank sum test.

Since *wake* alleles were previously reported to have reduced sleep and *nmf9* mice had abnormal circadian behaviors, we made related measurements for heterozygotes of our engineered null mutations. Consistent with previous reports, *wake* alleles showed decreased sleep in both sexes (Fig 9). While male null heterozygotes also showed reduced daily sleep, surprisingly, females showed slightly increased daily sleep. Null heterozygotes also had increased latencies to first sleep bout after lights on (daytime sleep latency), and heterozygote females had increased latency to first sleep bout after lights off (nighttime sleep latency) as well. Increased latencies were consistent with, but less severe than, *wake* alleles (Fig 9). Reduced sleep and increased sleep latencies could not be accounted for by an increase in rate of movement since heterozygotes for engineered null alleles showed moderately reduced waking activity levels, as did P-element *wake* alleles (Fig 8F). Statistical summaries for these tests are given in S5 Table. Together, these results show a strong requirement for *CG45058* function for viability, activity level, and sleep-related measures; show sexual dimorphism for impact on daily sleep; and resolve a conflict between previous reports of *CG45058* null phenotypes.

## Discussion

### Functional annotation of a novel gene genealogy

Through a combination of genetic and molecular approaches, we showed that *Nmf9* encodes a novel yet highly conserved protein that is functionally important to a distinct set of neurological phenotypes in both mice and flies. Starting with the mutation, we identified *Nmf9* transcripts using positional cloning and experimental validation of predicted exons. RT-PCR and in situ hybridization experiments defined timing and location of gene expression. Sites of expression predicted, in addition to readily observed vestibular abnormalities, substantial phenotypes in fear learning and circadian behavior that were not obvious from the initial description or gross observation. Sequence information from >100 animal homologs identified distinct regions of strong evolutionary constraint, including ANK and FN3 motifs and three novel segments that lack motif annotation. Induced mutations at most conserved peptide sequence among the novel conserved domains produced strong phenotypes in both mice and flies, including a single glycine to alanine substitution in mice that failed to complement the original *nmf9* allele. Among animal and non-animal Holozoan homologues some strongly predicted functional motifs appeared to be modular across phyla, including two small GTPase-binding motifs, CRIB and RA. Among animals, the Nmf9 homology group appears to have been lost only in urochordates, based on four genomes available in that taxon. The N-terminal CRIB domain was found only in choanoflagellate and filasterean sequences and was either gained in these lineages or lost in the lineage leading to animals. The RA domain appears to have been lost in a derived paralog during or soon after a gene duplication event at the basal vertebrate lineage and the ancestral paralog was lost in the basal mammalian lineage.

### Expression and function of *Nmf9* in mammalian brain

*Nmf9* showed a complex pattern of expression in the vestibular system, olfactory system, and regions of the brain implicated in satiety and metabolism, innate anxiety, fear learning, and circadian rhythm. We tested the functional integrity of each of these systems with a battery of standardized behavioral assays. Deficits in vestibular, circadian and fear learning measures were clear despite significant sex differences. Abnormalities in measures of anxiety and appetitive behavior were nominally significant, but confounded by hyperactivity in the mutant mice–particularly after sustained handling, which may suggest either an anxiety-related aspect or perhaps feedback from disturbed sensory input from the vestibular system. Although mutants and non-mutant littermates were easily distinguished by overall behavior, mutant phenotypes for any single formalized test were not fully penetrant and tended toward higher variance than control littermate values, which suggests that *Nmf9* may be important to the robustness of these pathways, but not essential to their basic function. On this interpretation, loss of *Nmf9* activity in AMY, LS, and the olfactory system may not be disruptive enough to these circuits for behavioral phenotypes to be detected on a standard inbred background, but might have consequences that would be subject to selection under the more competitive fitness constraints of wild populations. Although we observed striking *Nmf9* expression in cortical ventricular zones, the adult cortex appeared normal on gross inspection, with no obvious loss of cells in any of the *Nmf9*-enriched sites.

Females were more severely affected than males by the *nmf9* mutation in several tests (Figs 1 and 4). While both sexes express *Nmf9* RNA, we have not explicitly tested for quantitative differences in specific pattern elements. Sex-dependence is known for several mouse behaviors both at baseline and in response to perturbations [29]. Interestingly, mutations of *Ntrk2* (TrkB) also induce vestibular defects that are substantially more pronounced in female mice [30], although any shared mechanism remains to be explored.

### Functions of *CG45058* in *Drosophila*

Our analysis of *CG45058* in *Drosophila* resolves a conflict in the current literature and extends both sets of previous findings. While our work was nearing completion, two other groups reported putative null mutations in *CG45058*, but with very different primary outcomes. In a P-element screen for sleep phenotypes, Mark Wu and colleagues reported viable P-element and imprecise excision alleles, which they termed *wide-awake* (*wake*) based on increased latency to sleep and decreased total sleep by mutant animals [19]. In contrast, through an RNAi screen for regulators of asymmetric cell division, Juergen Knoblich and colleagues reported pupal-lethal gene deletion alleles of *CG45058*, which they termed *banderuola* (*bnd*) based on the cytological appearance of dividing sensory organ precursor cells [20]. A major difference between these studies is the nature of the alleles examined. *CG45058* has several annotated transcripts, which primarily differ in transcriptional start site and utilization of 5’ exons that are not well-conserved outside of Diptera. The *wake* alleles occur in the variant 5’ region of the gene and appear likely to affect only those transcripts, but not several others that include all of the conserved protein coding sequences. Alternatively, the *bnd* gene deletion allele might have removed regulatory sequences or barriers that influence expression of neighboring genes resulting in a synthetic phenotype. Our results resolve this conflict by creating discrete loss-of-function alleles induced at three distinct, functionally important sites in the predicted protein, encoded by separate exons that are each included in all known *CG45058* transcripts. These alleles strikingly reduced adult viability and locomotor function of surviving adults, demonstrating that this is the null phenotype, in support of Mauri et al. Heterozygotes for these null alleles had reduced waking activity, increased latencies to first sleep bout, and effects on total sleep, confirming and extending the behavioral results of Liu et al. for *wake* alleles. Our data show sexually dimorphic effects on total sleep in null heterozygotes, in contrast to decreased sleep found in both sexes of *wake* mutants. This allelic difference suggests either distinct cellular functions of *CG45058* isoforms or, perhaps more likely, isoform-specific expression in cell types that impinge on sleep regulation.

### Conservation, divergence, and pleiotropy

While the *Nmf9* orthology group shows extraordinary conservation–including highly constrained sequence domains that had no prior functional or motif annotation–the reorganization of putative GTPase-binding domains across major phylogenetic boundaries, loss of the homology group in urochordates, and duplication and rescission events in the vertebrate lineage all suggest a degree of adaptive plasticity that may be reflected in both shared and lineage-specific requirements. Indeed, mutations in the *Nmf9* homologues of both mouse and fly resulted in phenotypes related to daily cycles of activity, as well as locomotor abnormalities, but with some clear differences. While mouse *nmf9* null alleles showed hyperactivity and tremor that increased over the course of six months, fly mutations at distinct conserved domains (ANK, FN3, and conserved domain 2) produced severe locomotor retardation and early death, within one week of eclosion. These studies lay a foundation for understanding both common functions of *Nmf9* homologs and lineage-restricted activities that might relate to derived loss of the RA domain or other lineage-specific features. How differences in domain architecture and expression patterns reshape the functional networks in which Nmf9/CG45058 proteins act will surely be of interest in the evolutionary development of the nervous system.

## Materials and Methods

### *Nmf9* genetic mapping

Coisogenic C57BL/6J–*nmf9* mice were obtained from the Neuroscience Mutagenesis Facility (NMF) and AKR/J, BALB/cJ, C3H/HeJ and DBA/2J mapping partners from production colonies at the Jackson Laboratory. Conventional exclusion mapping was performed as described [11, 31]. PCR primers for new genetic markers are given in S1 Table. New markers were also typed on a smaller C57BL/6J–*nur12* x BALB/cJ cross obtained from NMF. C57BL/6J–*nmf9* mutant line was maintained on C57BL/6J and genotyped by sequencing.

### Gene expression detection and measurements

Northern blots were performed by standard methods [32], essentially as described [33]. RNA from whole brains was extracted with Trizol reagent (Life Technologies). Poly(A)^+^ RNA was isolated on Oligo(dT) cellulose type 7 (Amersham Biosciences). Concentrations and integrity were verified by spectrophotometry and gel electrophoresis. 8.5 μg poly(A)^+^ RNA per sample was electrophoresed through a denaturing formaldehyde/agarose gel and transferred to Hybond-N+ membrane (Thermo Fisher). Size standard was removed prior to transfer and imaged by ethidium bromide fluorescence. Probes were synthesized from two cloned fragments of *Nmf9* by random priming in the presence of ^32^P-dCTP and hybridized overnight. Blots were exposed to a phosphor screen for 5 days before quantitative imaging with a Storm 860 instrument (Molecular Dynamics). Quantitative RT-PCR was performed using SybrGreen fluorescence quantification on a BioRad CFX96 instrument. Expression was relative to *Gapdh* and *RP49* in *Drosophila*, and to *GAPDH* and *SDHA* in zebrafish.

### *In situ* hybridization

Mouse tissues were processed by a standard method [34] as previously modified [8]. Briefly, embryos were fixed in formalin, adult mouse brain in 4% PFA. Samples were cryoprotected in 30% sucrose and sectioned at 20 μm. Slides were treated 15’ with boiling sodium citrate, followed by acetic anhydride in triethanolamine prior to hybridization with dioxigenin-labeled RNA probes at 65°C overnight. After hybridization and washing, sections were blocked with 5% normal donkey serum (Jackson ImmunoResearch) in PBSTX (0.2% Triton-X100 in PBS) for 1 hour and incubated with anti-digoxigenin-AP Fab fragments (Roche) at 1:2000 overnight at 4°C. Whole mount *in situ* in zebrafish was performed as described [35]. Samples were fixed in 4% PFA. Proteinase K was used for antigen retrieval at 10 μg/mL for 1 hour at 37°C. Hybridization was at 65°C for 48 hours. Samples were blocked with 2% normal donkey serum, 2mg/mL BSA (NEB) in PBTx and incubated with 1:5000 anti-dig-AP Fab fragments overnight at 4°C. Hybridization to whole mount fly embryos was performed as described [36]. Briefly, embryos were fixed in methanol and treated with Proteinase K at 37C for 7 minutes. Embryos were hybridized at 55°C overnight. Samples were blocked with 1:10 Western Blocking Reagent (Roche) in PBTwx (0.1% Tween 20 and 0.1% Triton-X in PBS) and incubated with 1:1000 anti-dig-AP Fab fragments for 2 hours at 4°C. All probes were prepared by in vitro transcription from linearized plasmid templates and diluted in hybridization buffer prior to use. Sea anemone in situ hybridization was performed as described [37]. Probe templates for all species were generated by RT-PCR and cloned into appropriate vectors for in vitro transcription.

### Mouse behavioral assays

All behavioral tests were performed on mice between 2 to 6 months of age, with wild-type littermates as control to the mutant animals, by investigators blinded to the animals’ genotypes.

#### Vestibular function

For the forced swim test, animals were individually placed in a 4L beaker filled with 3L 25°C water and the swim time measured as the length of time an animal was able to keep its snout above water [38]. For the landing test, animals were individually lifted by their tails. Normal animals extended their head and forelimbs toward the ground, exhibiting landing behavior, whereas mutant animals tend to curl their trunk sideways or ventrally and occasionally trying to rotate [39]. The presence of the curling / rotation behavior was scored. For nodding, tremor, circling, and hyperactivity, each animal was placed in a fresh cage and observed for one minute to score each phenotype.

#### Auditory function

The startle response was performed in startle chambers (SR-Lab, San Diego Instruments) in the La Jolla Neuroscience Blueprint Behavioral Core Facility at The Scripps Research Institute (TSRI Behavioral Core). The chambers consisted of nonrestrictive Plexiglas cylinders 5 cm in diameter resting on a Plexiglas platform in a ventilated chamber. High-frequency speakers mounted 33 cm above the cylinders produced all acoustic stimuli, which were controlled by SR-LAB software. Piezoelectric accelerometers mounted under the cylinders transduce movements of the animal, which were digitized and stored by an interface and computer assembly. Startle pulses started at 70 dB, 40ms in duration, and increased to 120 dB in 5 dB increments and then ramped back down, interspersed by no stimulus trials. The background noise level was at 70 dB.

#### Fear learning

Conditioning was performed by the TSRI Behavioral Core and took place in Freeze Monitor chambers (Med Associates) stationed in sound-proof boxes. The Plexiglas conditioning chambers (26 x 26 x 17 cm) had speakers and lights mounted on two opposite walls and were installed with an electric grid floor. On day 1, mice were habituated in the conditioning chamber for five minutes. On day 2, mice were exposed to the context and conditioned stimulus (30 seconds, 3000 Hz, 80 dB sound) in association with foot shock (0.70 mA, 2 second, scrambled current). Each mouse received 2 shock exposures, each in the last 2 seconds of a 30 second tone exposure during a 5.5 minute session. On day 3, contextual conditioning in animals was measured in a 5 minute test in the training chamber. On day 4, cued conditioning was measured in a novel context, where the chamber previously used was disguised (new opaque plastic rounded compartment replacing old clear square compartment, new opaque plastic floor replacing metal grid, novel odor with a drop of orange extract under floor). The animals were allowed to habituate for 3 minutes before exposure to the conditioned stimulus (tone) for 3 minutes. Freezing behavior was measured in all sessions by a validated computer-controlled recording of photocell beam interruptions [40].

#### Circadian function

Mutant and wildtype littermates of either sex, between three-five months of age, were individually housed with food and water *ad libitum*. Activity records were plotted as actograms and analyzed using ClockLab (Actimetrics, Evanston, IL). Unless otherwise noted, all calculations were performed across an interval of approximately 14 days. The period estimates were analyzed with the activity onset predicted by ClockLab, after manually checking and adjusting when necessary. Onset tau and onset error of the period of daily activity in DD were calculated using the adjusted activity onset data. Power and tau in LD 12:12 and DD were additionally determined with Chi-square periodogram analysis.

#### Innate anxiety

The elevated plus-maze and open field tests were performed at the TSRI Behavioral Core. The plus maze apparatus had four arms (5 x 30 cm) at right angles and was elevated 30 cm from the floor. The two closed arms had 16 cm high walls. The two open arms had a 0.5 cm lip and no walls. Animals were placed in the center of the maze and allowed free access to all arms for 5 minutes. Behaviors were recorded with mounted cameras. The open field apparatus was a 50 x 50 cm plexiglass square surrounded by 22 cm high walls. The field was divided into 16 squares (12 outer and 4 inner) of equal areas. Animals were placed in the center of the field and recorded for 10 to 60 minute with mounted cameras. The marble burying test was performed by placing individual animals in a standard mouse cage containing 5 cm deep bedding with 20 small marbles arranged in an evenly spaced grid of 4 rows of 5 marbles on top of the bedding material. After 30 min mice were removed and the number of marbles that were at least 2/3 covered by bedding was counted.

#### Olfaction

The buried food finding test was performed on individually housed mice. Beginning 3 days before testing, animals received one piece of Froot Loops cereal (Kellog's, the same color/flavor was used for all animals) per day with *ad libitum* access to water and normal chow. Fourteen hours (overnight) before testing, animals were deprived of food while water remained ad libitum. Each animal was tested in a clean cage containing 2 cm of bedding, in the corner opposite to the corner where a piece of cereal was hidden. The time taken to locate and hold the cereal in both of its paws was recorded with a stopwatch. The odor habituation / dishabituation test was performed by placing individual animals in a clean cage and allowing 30 minutes for habituation. Non-social odors were presented before social odors. For non-social odors, a cotton-tipped applicator dipped in either de-ionized water or 1:100 dilution of almond or vanilla extract (McCormick &Co) was presented. For social odors, each applicator was used to swab the inside of a cage that had housed a single non-littermate animal for three days and then presented to the animal being tested. The length of time each animal spent investigating the tip applicator was recorded, with two minutes given per applicator trial. The same odor was presented in three consecutive trials, with the same order of odor presentation for all animals.

#### Satiety and metabolism

Animals were weighed at 21 days, 2 months, and 6 months of age. For food consumption test, a known mass of food was provided to the animals *ad libitum* at the start of experiment. The mass of the food was weighed each subsequent evening at the same time and monitored for spillage (Huszar et al. 1997, Crawley 1999).

### Conservation analysis

Protein sequences homologous to mouse Nmf9 were retrieved through NCBI tblastn, UCSC Genome Browser, Ensembl, and JGI web sites and made use of GNOMON and ENSEMBL transcript predictions. Identified fragments were used to search the full length sequences in surrounding genomic sequences where available (see S4 Table). For homologs inferred purely through genomic sequences, a combination of BLAT [41], BLAST [42] and GENSCAN [43] programs were used to predict open reading frames. All database searches were performed before February 2013. For the full-length protein analysis, the best-annotated sequence of each evolutionary branch was used. For the high resolution analysis sequences with more than 50% gaps in that domain were excluded. Predicted cDNA sequences were translated using ExPASy Translate [44] and aligned using MUSCLE [45, 46] and the result of the amino acid alignment used to manually correct the nucleic acid alignment. Conservation rates were calculated with Datamonkey [47] using codon data type and universal genetic code with neighbor joining tree. Motif search was performed with Motif Scan [48], Scansite 2.0 Scan Motif [49], Scan Prosite (ExPAsy), InterProScan 4 [50], Pfam [51], and SMART 6 [52]. Protein modification scan was performed using The Sulfinator (ExPASy) for sulfination and YinOYang [53] for glycosylation and phosphorylation.

### Genome edited animals

Genome-edited mice were generated essentially as described [17, 28]. Briefly, in-vitro synthesized Cas9 mRNA, sgRNA, and ssDNA homology-directed repair oligos were co-injected as a cocktail into C57BL/6 one-cell embryos at the Moores UCSD Cancer Center Transgenic Mouse Shared Resource. Oligonucleotide sequences are listed in S6 Table. sgRNA templates and ssDNA repair oligonucleotides were synthesized as Ultramers by IDT. All procedures were approved by the UCSD IACUC. RNA and DNA reagents for fly injection were prepared as above. Conserved Domain 2 mutants were generated by co-injection of in-vitro synthesized Cas9, sgRNA, and ssDNA repair oligo into *w* ^*1118*^ embryos. ANK and FN3 mutants were generated by co-injection of sgRNA and repair oligonucleotides into Cas9-expressing embryos (PBac<y[+mDint2] = vas-Cas9>VK00027, Bloomington Stock Number 51324) as described by [54]. All fly embryo injections were performed by Rainbow Transgenic Flies, Inc. (Camarillo, CA). Oligonucleotide sequences are listed in S6 Table.

### *Drosophila* stocks

Mutations were verified by Sanger sequencing of PCR products encompassing targeted genomic loci. Transposon mutant and deficiency lines for CG45058 were obtained from the Bloomington Stock Center according to the following stock numbers: *wake* ^*EY02219*^ [15858], *wake* ^*KG02188*^ [14082], *wake* ^*KG08407*^ [15129], *wake* ^*MI02905*^ [37162], Df(3R)Exel6273 [7740], Df(3R)ED6085 [150049], Df(3R)ED6091 [9092], Df(3R)BSC527 [25055], Df(3R)Exel6192 [7671], Df(3R)BSC618 [25693], and Df(3R)ED6090 [150614].

### *Drosophila* behavior assays

Flies were raised at room temperature (~21°C) on standard cornmeal/molasses. Within 1–2 days of eclosion, flies were assayed for wing orientation and the ability to maintain a standing position. For sleep assays, 1–5 day old flies were loaded into 65x5 mm glass tubes containing 5% sucrose and 2% agarose and entrained to a 12hr:12hr light:dark (LD) cycle for 2 days before recording sleep/wake patterns using the *Drosophila* Activity Monitoring System (Trikinetics, Waltham, MA). Sleep was defined as 5 minutes of inactivity and, along with waking activity, was measured at 25°C using custom software as previously described [55].

### Ethics statement

Mice were euthanized by CO2 inhalation or by perfusion or organ removal under deep anesthesia with tribromoethanol (avertin). Fish embryos were euthanized by fixation or snap-frozen in E3 media. All vertebrate animal procedures were approved by the University of California San Diego Institutional Animal Care and Use Committee (IACUC). The University of California San Diego is AAALAC accredited, AAALAC institutional number 000503.

## Supporting Information

S1 Table Genetic markers used for genetic fine mapping. Dinucleotide repeat sequences were identified from an early draft public mouse reference sequence (MGSCv2) and tested for length polymorphism in mapping strains.
(XLSX)

S2 Table Statistical analyses of mouse behavioral tests. (XLSX)

S3 Table Circadian phenotype of *Nmf9*. Animals were housed in 12:12 LD cycles for a minimum of 7 days prior to transition to DD. Tau, power, and onset were calculated as average over ~14 days. N = 14 +/+ females, 12 mutant females, 9 +/+ males, 10 mutant males. Data was analyzed using two-way ANOVA.
(XLSX)

S4 Table Species used in evolution conservation analysis. Homologs of *Nmf9* were identified in various databases and hand curated. Due to quality of genomes available, sequences that contain more than 50% gaps were removed and not included in this list. Due to poor assembly, all homolog sequences, with the exception of *H*. *sapiens*, was manually assembled utilizing the BLAST, BLAST, TBLASTN tools available in many of the databases, the ID of the seed sequence was listed when available. Species highlighted in yellow indicate the sequences used on whole-protein conservation analysis. 52 species containing ancestral versions of the homolog and 61 species containing derived version of the homolog were used for the region specific analysis of conservation.
(XLSX)

S5 Table Statistical analyses of *Drosophila* behavioral tests. (XLSX)

S6 Table Oligonucleotides used in genome editing of flies and mice. (XLSX)

S1 Fig Mutant animals have normal inner ear histology and hearing. (A) Ampulla, saccule, Scarpa's ganglion, utricle, and semi-circular canals were grossly normal in *nmf9* animals. (B) Acoustic brainstem responses were within normal range (waveform threshold ≤25 dB) for 5 of 6 mutant mice between 2–4 months age. (C) Acoustic startle responses were also normal in a separate cohort animals between 2–6 months age. Two-factor MANOVA p = 0.30 for genotype, p = 0.28 for sex. N = 12 +/+ females, 12 +/+ males, 13 mutant females, 7 mutant males. Error bars, s.e.m.
(TIF)

S2 Fig *Nmf9* mRNA includes both *Ankfn1* and *4932411E22Rik*. (A) UCSC Genome Browser shows minus-strand transcripts for the 3’ exons of *Ankfn1* and 5’ exons of *E22* compared to BLAT alignment of empirically determined *Nmf9* mRNA sequence. Positions of primers used for RT-PCR are indicated. (B) Relative quantities for real-time fluorescence measurements (SYBR) of RT-PCR products from the indicated assays from either brain cDNA or cloned plasmid. (C) Publicly deposited cDNA sequences for insect (*D*. *melanogaster*, A. mellifera), echinoderm (*S*. *purpuratus*), nematode (*C*. *elegans*) and cnidarian (*N*. *vectensis*) also support a single transcript across segments homologous to *Ankfn1* and *E22*. (D) In situ hybridization patterns were indistinguishable for *Ankfn1* and *E22* probes in ventromedial hypothalamus (VMH), amygdala (AMY), lateral septum (LS), piriform cortex (PC), suprachiasmatic nuclei (SCN) and spinal cord (SpC). This mirrors data for both annotations in the Allen Brain Atlas (Fig 3B).
(TIF)

S3 Fig *Nmf9* expression in developing brain and sensory structures. *Nmf9* RNA was detected by *in situ* hybridization as early as E14.5 in ventricular zones, inner ear, and nasal epithelium.
(TIF)

S4 Fig Modular organization of *Nmf9* homologs. Known motifs are indicated in green. Highly consereved domains novel to the *Nmf9* homology group are shown in blue. An aminoterminal CRIB domain was found only in the two choanoflagellae species, while inclusion or loss of a ras-association domain (RA) appears not to be monophyletic. Vertebrate species basal to mammals had paralogous copies, one with and one without the RA domain in each species. All mammals examined had only one copy, which never had an identifiable RA domain. Conservation of the first ANK repeat, and consensus match to the motif definition were poor or absent in some invertebrate and choanozoa species.
(TIF)

S5 Fig Core *Nmf9* domains have lineage-dependent patterns of conservation. High-resolution sliding window analysis of domains, with inclusion of species that lacked full-length sequence. Separate analyses were performed for ancestral homologs and vertebrate-derived homologs. Y-axis shows dN/dS values calculated using HyPhy [56]. The 10 residues with smallest dN in each analysis are plotted in orange. Light blue points indicate gaps in sequence. Amino acid residues are given for regions of highest conservation. (A) Annotated ankyrin and fibronection type III motifs are plotted. For the ankyrin repeats, N = 53 species for derived homologs, 47 for ancestral homologs. For fibronectin type III repeats, N = 50 derived, 50 ancestral homologs. (B) The three highly conserved domains that do not align with known motif definitions are plotted. For domain 1, N = 60 species for derived homologs, 52 for ancestral. For domain 2, N = 54 derived, 49 ancestral. For domain 3, 49 derived, 48 ancestral.
(TIF)

S6 Fig Expression of *Nmf9* homologs suggests both conserved and adapted functions. *Nmf9* homologues were expressed during development in (A) fruit fly and (B) zebrafish. Samples included 3 biological replicates for fruit fly, but only technical replicates of single samples for each zebrafish stage and paralogous gene. (C) *In situ* hybridization to *Drosophila* embryos showed broad *CG45058* expression, including CNS, agreeing with stage-specific expression in the BDGP in situ database (http://insitu.fruitfly.org/). (D) Expression in sea anemone *N*. *vectensis* planulae (left) includes endoderm (arrow) and some apical tuft cells. In polyps, expression appears uniform throughout the endoderm (arrow) and mesenteries. (E) In zebrafish embryos, ancestral paralog retained a broad but weak expression pattern that includes expression in the developing inner ear (box and inset). The derived paralog showed a cleaner, more restricted pattern.
(TIF)

S7 Fig *CG45058* allele locations and deficiency mapping for severe phenotypes. (A) Schematic of the *CG45058* locus shows exon position of encoded protein domains (blue) across transcripts (navy) from the UCSC browser. Chromatin accesibility to DNase I at stage 5 (green), 9 (orange), 10 (red), 11(blue) and 14 (purple) embryos suggests dynamic regulation of alternative transcripts. (B) UCSC transcripts (navy) in the interval around *CG45058* (red) on *Drosophila* chromosome 3R are shown relative to deficiencies that complement (green bars) or fail to complement (red bars) frameshift mutation at conserved domain 2 (GLYLGYLK). The inferred interval (yellow box) defined by the shared breakpoint of Df(3R)Exel6273 and Df(3R)Exel6192 and the right breakpoint of Df(3R)ED6090 contains parts of 6 transcription units.
(TIF)

S1 Video Comparison of homozygous *nmf9* female to littermate control on Coplin jars at 10 months of age shows tremor, tilted head posture and head nodding in the mutant. Behavior in the home cage shows circling by a strongly affected mutant female compared to non-circling littermate control.
(MOV)

S2 Video Trans-heterzyogotes for the G-to-A edited and *nmf9* chemically induced alleles show agitation, tremor and mild vestibular defects compared to same-sex littermates that are heterozygous for either mutant allele over wild type. Birth date and identifier is indicated for each animal.
(MP4)

## References

[1] Beutler B, Du X, Xia Y. Precis on forward genetics in mice. Nat Immunol. 2007;8(7):659–64. Epub 2007/06/21. 17579639 10.1038/ni0707-659

[2] Justice MJ, Noveroske JK, Weber JS, Zheng B, Bradley A. Mouse ENU mutagenesis. Hum Mol Genet. 1999;8(10):1955–63. Epub 1999/09/02. 10469849 10.1093/hmg/8.10.1955

[3] Clark AT, Goldowitz D, Takahashi JS, Vitaterna MH, Siepka SM, Peters LL, et al Implementing large-scale ENU mutagenesis screens in North America. Genetica. 2004;122(1):51–64. Epub 2004/12/29. 15619961 10.1007/s10709-004-1436-6 PMC3774779

[4] Concepcion D, Seburn K, Wen G, Frankel WN, Hamilton BA. Mutation rate and predicted phenotypic target sizes in ENU-treated mice. Genetics. 2004;168(2):953–9. 15514066 10.1534/genetics.104.029843 PMC1448829

[5] Kawai J, Shinagawa A, Shibata K, Yoshino M, Itoh M, Ishii Y, et al Functional annotation of a full-length mouse cDNA collection. Nature. 2001;409(6821):685–90. Epub 2001/02/24. 11217851 10.1038/35055500

[6] Okazaki Y, Furuno M, Kasukawa T, Adachi J, Bono H, Kondo S, et al Analysis of the mouse transcriptome based on functional annotation of 60,770 full-length cDNAs. Nature. 2002;420(6915):563–73. 12466851 10.1038/nature01266

[7] Alcaraz WA, Chen E, Valdes P, Kim E, Lo YH, Vo J, et al Modifier genes and non-genetic factors reshape anatomical deficits in Zfp423-deficient mice. Hum Mol Genet. 2011;20(19):3822–30. Epub 2011/07/07. 10.1093/hmg/ddr300 21729880 PMC3168291

[8] Alcaraz WA, Gold DA, Raponi E, Gent PM, Concepcion D, Hamilton BA. Zfp423 controls proliferation and differentiation of neural precursors in cerebellar vermis formation. Proc Natl Acad Sci U S A. 2006;103(51):19424–9. 17151198 10.1073/pnas.0609184103 PMC1748242

[9] Gold DA, Baek SH, Schork NJ, Rose DW, Larsen DD, Sachs BD, et al RORa coordinates reciprocal signaling in cerebellar development through sonic hedgehog and calcium-dependent pathways. Neuron. 2003;40(6):1119–31. 14687547 10.1016/s0896-6273(03)00769-4 PMC2717708

[10] Hamilton BA, Frankel WN, Kerrebrock AW, Hawkins TL, FitzHugh W, Kusumi K, et al Disruption of the nuclear hormone receptor RORa in *staggerer* mice. Nature. 1996;379:736–9. 8602221 10.1038/379736a0

[11] Hamilton BA, Smith DJ, Mueller KL, K A.W., B R.T., van Berkel V, et al The *vibrator* mutation causes neurodegeneration via reduced expression of PITPa: positional complementation cloning and extragenic suppression. Neuron. 1997;18:711–22. 9182797 10.1016/s0896-6273(00)80312-8

[12] Bassett AR, Tibbit C, Ponting CP, Liu JL. Highly efficient targeted mutagenesis of *Drosophila* with the CRISPR/Cas9 system. Cell Rep. 2013;4(1):220–8. Epub 2013/07/06. 10.1016/j.celrep.2013.06.020 23827738 PMC3714591

[13] Cong L, Ran FA, Cox D, Lin S, Barretto R, Habib N, et al Multiplex genome engineering using CRISPR/Cas systems. Science. 2013;339(6121):819–23. Epub 2013/01/05. 10.1126/science.1231143 23287718 PMC3795411

[14] Gratz SJ, Cummings AM, Nguyen JN, Hamm DC, Donohue LK, Harrison MM, et al Genome engineering of *Drosophila* with the CRISPR RNA-guided Cas9 nuclease. Genetics. 2013;194(4):1029–35. Epub 2013/05/28. 10.1534/genetics.113.152710 23709638 PMC3730909

[15] Jinek M, East A, Cheng A, Lin S, Ma E, Doudna J. RNA-programmed genome editing in human cells. Elife. 2013;2:e00471 Epub 2013/02/07. 10.7554/eLife.00471 23386978 PMC3557905

[16] Mali P, Yang L, Esvelt KM, Aach J, Guell M, DiCarlo JE, et al RNA-guided human genome engineering via Cas9. Science. 2013;339(6121):823–6. Epub 2013/01/05. 10.1126/science.1232033 23287722 PMC3712628

[17] Wang H, Yang H, Shivalila CS, Dawlaty MM, Cheng AW, Zhang F, et al One-step generation of mice carrying mutations in multiple genes by CRISPR/Cas-mediated genome engineering. Cell. 2013;153(4):910–8. Epub 2013/05/07. 10.1016/j.cell.2013.04.025 23643243 PMC3969854

[18] Yu Z, Ren M, Wang Z, Zhang B, Rong YS, Jiao R, et al Highly efficient genome modifications mediated by CRISPR/Cas9 in *Drosophila* . Genetics. 2013;195(1):289–91. Epub 2013/07/09. 10.1534/genetics.113.153825 23833182 PMC3761309

[19] Liu S, Lamaze A, Liu Q, Tabuchi M, Yang Y, Fowler M, et al WIDE AWAKE mediates the circadian timing of sleep onset. Neuron. 2014;82(1):151–66. Epub 2014/03/19. 10.1016/j.neuron.2014.01.040 24631345 PMC3982794

[20] Mauri F, Reichardt I, Mummery-Widmer JL, Yamazaki M, Knoblich JA. The conserved discs-large binding partner Banderuola regulates asymmetric cell division in *Drosophila* . Curr Biol. 2014;24(16):1811–25. Epub 2014/08/05. 10.1016/j.cub.2014.06.059 25088559

[21] Dietrich WF, Miller J, Steen R, Merchant MA, Damron-Boles D, Husain Z, et al A comprehensive genetic map of the mouse genome. Nature. 1996;380(6570):149–52. 8600386 10.1038/380149a0

[22] Keays DA, Clark TG, Flint J. Estimating the number of coding mutations in genotypic- and phenotypic-driven N-ethyl-N-nitrosourea (ENU) screens. Mamm Genome. 2006;17(3):230–8. Epub 2006/03/07. 16518690 10.1007/s00335-005-0101-4

[23] Bermingham NA, Hassan BA, Price SD, Vollrath MA, Ben-Arie N, Eatock RA, et al Math1: an essential gene for the generation of inner ear hair cells. Science. 1999;284(5421):1837–41. Epub 1999/06/12. 10364557 10.1126/science.284.5421.1837

[24] Kelley MW. Hair cell development: commitment through differentiation. Brain Res. 2006;1091(1):172–85. Epub 2006/04/22. 16626654 10.1016/j.brainres.2006.02.062

[25] Lein ES, Hawrylycz MJ, Ao N, Ayres M, Bensinger A, Bernard A, et al Genome-wide atlas of gene expression in the adult mouse brain. Nature. 2007;445(7124):168–76. Epub 2006/12/08. 17151600 10.1038/nature05453

[26] Pond SL, Frost SD. Datamonkey: rapid detection of selective pressure on individual sites of codon alignments. Bioinformatics. 2005;21(10):2531–3. Epub 2005/02/17. 15713735 10.1093/bioinformatics/bti320

[27] Marlow HQ, Srivastava M, Matus DQ, Rokhsar D, Martindale MQ. Anatomy and development of the nervous system of *Nematostella vectensis*, an anthozoan cnidarian. Dev Neurobiol. 2009;69(4):235–54. Epub 2009/01/27. 10.1002/dneu.20698 19170043

[28] Concepcion D, Ross KD, Hutt KR, Yeo GW, Hamilton BA. Nxf1 natural variant E610G is a semi-dominant suppressor of IAP-induced RNA processing defects. PLoS Genet. 2015;11(4):e1005123 Epub 2015/04/04. 10.1371/journal.pgen.1005123 25835743 PMC4383553

[29] Crawley JN. What's wrong with my mouse?: behavioral phenotyping of transgenic and knockout mice 2nd ed. Hoboken, New Jersey: John Wiley & Sons, Inc.; 2007.

[30] Lucas EK, Jegarl A, Clem RL. Mice lacking TrkB in parvalbumin-positive cells exhibit sexually dimorphic behavioral phenotypes. Behav Brain Res. 2014;274:219–25. Epub 2014/08/17. 10.1016/j.bbr.2014.08.011 . 25127683 PMC5063750

[31] Floyd JA, Gold DA, Concepcion D, Poon TH, Wang X, Keithley E, et al A natural allele of Nxf1 suppresses retrovirus insertional mutations. Nat Genet. 2003;35(3):221–8. 14517553 10.1038/ng1247 PMC2756099

[32] Sambrook J, Fritsch EF, Maniatis T. Molecular Cloning: A Laboratory Manual 2nd ed. Cold Spring Harbor, NY: Cold Spring Harbor Laboratory Press; 1989.

[33] Concepcion D, Flores-Garcia L, Hamilton BA. Multipotent genetic suppression of retrotransposon-induced mutations by Nxf1 through fine-tuning of alternative splicing. PLoS Genet. 2009;5(5):e1000484 Epub 2009/05/14. 10.1371/journal.pgen.1000484 19436707 PMC2674570

[34] Wilkinson DG, Nieto MA. Detection of messenger RNA by in situ hybridization to tissue sections and whole mounts. Methods Enzymol. 1993;225:361–73. Epub 1993/01/01. 8231863 10.1016/0076-6879(93)25025-w

[35] Clements WK, Kimelman D. LZIC regulates neuronal survival during zebrafish development. Dev Biol. 2005;283(2):322–34. Epub 2005/06/04. 15932753 10.1016/j.ydbio.2005.04.026

[36] Bergson C, McGinnis W. An autoregulatory enhancer element of the *Drosophila* homeotic gene Deformed. EMBO J. 1990;9(13):4287–97. Epub 1990/12/01. 1979945 10.1002/j.1460-2075.1990.tb07877.x PMC552211

[37] Wolenski FS, Layden MJ, Martindale MQ, Gilmore TD, Finnerty JR. Characterizing the spatiotemporal expression of RNAs and proteins in the starlet sea anemone, *Nematostella vectensis* . Nat Protoc. 2013;8(5):900–15. Epub 2013/04/13. 10.1038/nprot.2013.014 23579779 PMC4792812

[38] Paffenholz R, Bergstrom RA, Pasutto F, Wabnitz P, Munroe RJ, Jagla W, et al Vestibular defects in head-tilt mice result from mutations in Nox3, encoding an NADPH oxidase. Genes Dev. 2004;18(5):486–91. Epub 2004/03/12. 15014044 10.1101/gad.1172504 PMC374230

[39] Lessenich A, Lindemann S, Richter A, Hedrich HJ, Wedekind D, Kaiser A, et al A novel black-hooded mutant rat (ci3) with spontaneous circling behavior but normal auditory and vestibular functions. Neuroscience. 2001;107(4):615–28. Epub 2001/11/27. 11720785 10.1016/s0306-4522(01)00390-6

[40] Contarino A, Baca L, Kennelly A, Gold LH. Automated assessment of conditioning parameters for context and cued fear in mice. Learn Mem. 2002;9(2):89–96. Epub 2002/05/07. 11992019 10.1101/lm.43002 PMC155931

[41] Kent WJ. BLAT—the BLAST-like alignment tool. Genome Res. 2002;12(4):656–64. Epub 2002/04/05. Article published online before March 2002. 11932250 10.1101/gr.229202 PMC187518

[42] Altschul SF, Gish W, Miller W, Myers EW, Lipman DJ. Basic local alignment search tool. J Mol Biol. 1990;215(3):403–10. Epub 1990/10/05. 2231712 10.1016/S0022-2836(05)80360-2

[43] Burge C, Karlin S. Prediction of complete gene structures in human genomic DNA. J Mol Biol. 1997;268(1):78–94. Epub 1997/04/25. 9149143 10.1006/jmbi.1997.0951

[44] Artimo P, Jonnalagedda M, Arnold K, Baratin D, Csardi G, de Castro E, et al ExPASy: SIB bioinformatics resource portal. Nucleic Acids Res. 2012;40(Web Server issue):W597–603. Epub 2012/06/05. 10.1093/nar/gks400 22661580 PMC3394269

[45] Edgar RC. MUSCLE: a multiple sequence alignment method with reduced time and space complexity. BMC Bioinformatics. 2004;5:113 Epub 2004/08/21. 15318951 10.1186/1471-2105-5-113 PMC517706

[46] Edgar RC. MUSCLE: multiple sequence alignment with high accuracy and high throughput. Nucleic Acids Res. 2004;32(5):1792–7. Epub 2004/03/23. 15034147 10.1093/nar/gkh340 PMC390337

[47] Pond SL, Scheffler K, Gravenor MB, Poon AF, Frost SD. Evolutionary fingerprinting of genes. Mol Biol Evol. 2010;27(3):520–36. Epub 2009/10/30. 10.1093/molbev/msp260 19864470 PMC2877558

[48] Pagni M, Ioannidis V, Cerutti L, Zahn-Zabal M, Jongeneel CV, Hau J, et al MyHits: improvements to an interactive resource for analyzing protein sequences. Nucleic Acids Res. 2007;35(Web Server issue):W433–7. Epub 2007/06/05. 17545200 10.1093/nar/gkm352 PMC1933190

[49] Obenauer JC, Cantley LC, Yaffe MB. Scansite 2.0: Proteome-wide prediction of cell signaling interactions using short sequence motifs. Nucleic Acids Res. 2003;31(13):3635–41. Epub 2003/06/26. 12824383 10.1093/nar/gkg584 PMC168990

[50] Quevillon E, Silventoinen V, Pillai S, Harte N, Mulder N, Apweiler R, et al InterProScan: protein domains identifier. Nucleic Acids Res. 2005;33(Web Server issue):W116–20. Epub 2005/06/28. 15980438 10.1093/nar/gki442 PMC1160203

[51] Finn RD, Mistry J, Tate J, Coggill P, Heger A, Pollington JE, et al The Pfam protein families database. Nucleic Acids Res. 2010;38(Database issue):D211–22. Epub 2009/11/19. 10.1093/nar/gkp985 19920124 PMC2808889

[52] Letunic I, Doerks T, Bork P. SMART 6: recent updates and new developments. Nucleic Acids Res. 2009;37(Database issue):D229–32. Epub 2008/11/04. 10.1093/nar/gkn808 18978020 PMC2686533

[53] Gupta R, Brunak S. Prediction of glycosylation across the human proteome and the correlation to protein function. Pac Symp Biocomput. 2002:310–22. Epub 2002/04/04. 11928486

[54] Gratz SJ, Ukken FP, Rubinstein CD, Thiede G, Donohue LK, Cummings AM, et al. Highly Specific and Efficient CRISPR/Cas9-Catalyzed Homology-Directed Repair in *Drosophila*. Genetics. 2014. Epub 2014/01/31. 10.1534/genetics.113.160713 PMC3982687 24478335

[55] Wu M, Robinson JE, Joiner WJ. SLEEPLESS is a bifunctional regulator of excitability and cholinergic synaptic transmission. Curr Biol. 2014;24(6):621–9. Epub 2014/03/13. 10.1016/j.cub.2014.02.026 24613312 PMC4059605

[56] Pond SL, Frost SD, Muse SV. HyPhy: hypothesis testing using phylogenies. Bioinformatics. 2005;21(5):676–9. Epub 2004/10/29. 15509596 10.1093/bioinformatics/bti079

