# Supplementary figures and images for: Nmf9 Encodes a Highly Conserved Protein Important to Neurological Function in Mice and Flies
Source: PLoS Genet. 2015 Jul 1;11(7):e1005344. doi: 10.1371/journal.pgen.1005344 (PMC4488434; doi:10.1371/journal.pgen.1005344)

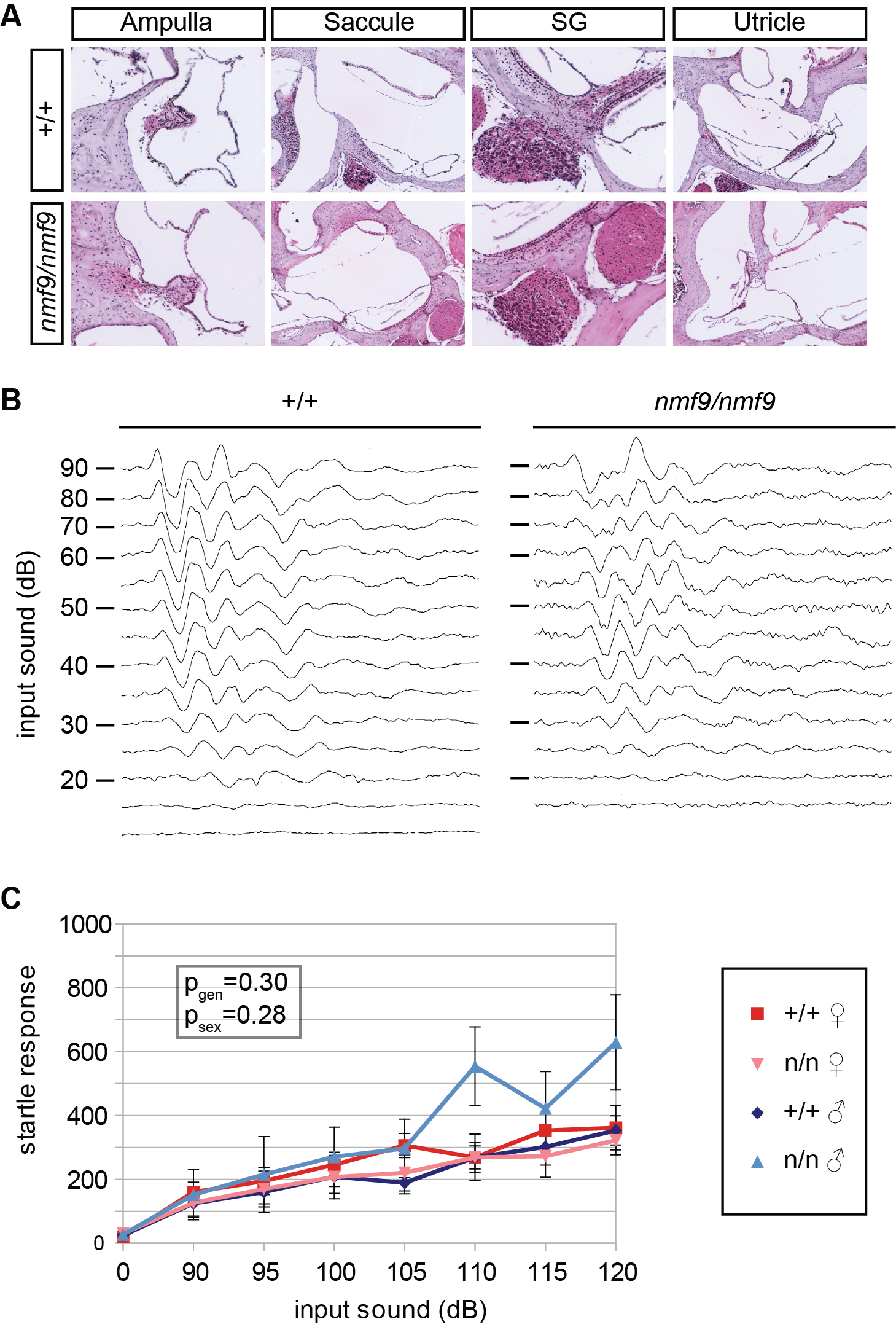

Supplement: S1 Fig — (A) Ampulla, saccule, Scarpa's ganglion, utricle, and semi-circular canals were grossly normal in nmf9 animals. (B) Acoustic brainstem responses were within normal range (waveform threshold ≤25 dB) for 5 of 6 mutant mice between 2–4 months age. (C) Acoustic startle responses were also normal in a separate cohort animals between 2–6 months age. Two-factor MANOVA p = 0.30 for genotype, p = 0.28 for sex. N = 12 +/+ females, 12 +/+ males, 13 mutant females, 7 mutant males. Error bars, s.e.m. (TIF) [file pgen.1005344.s007.tif]

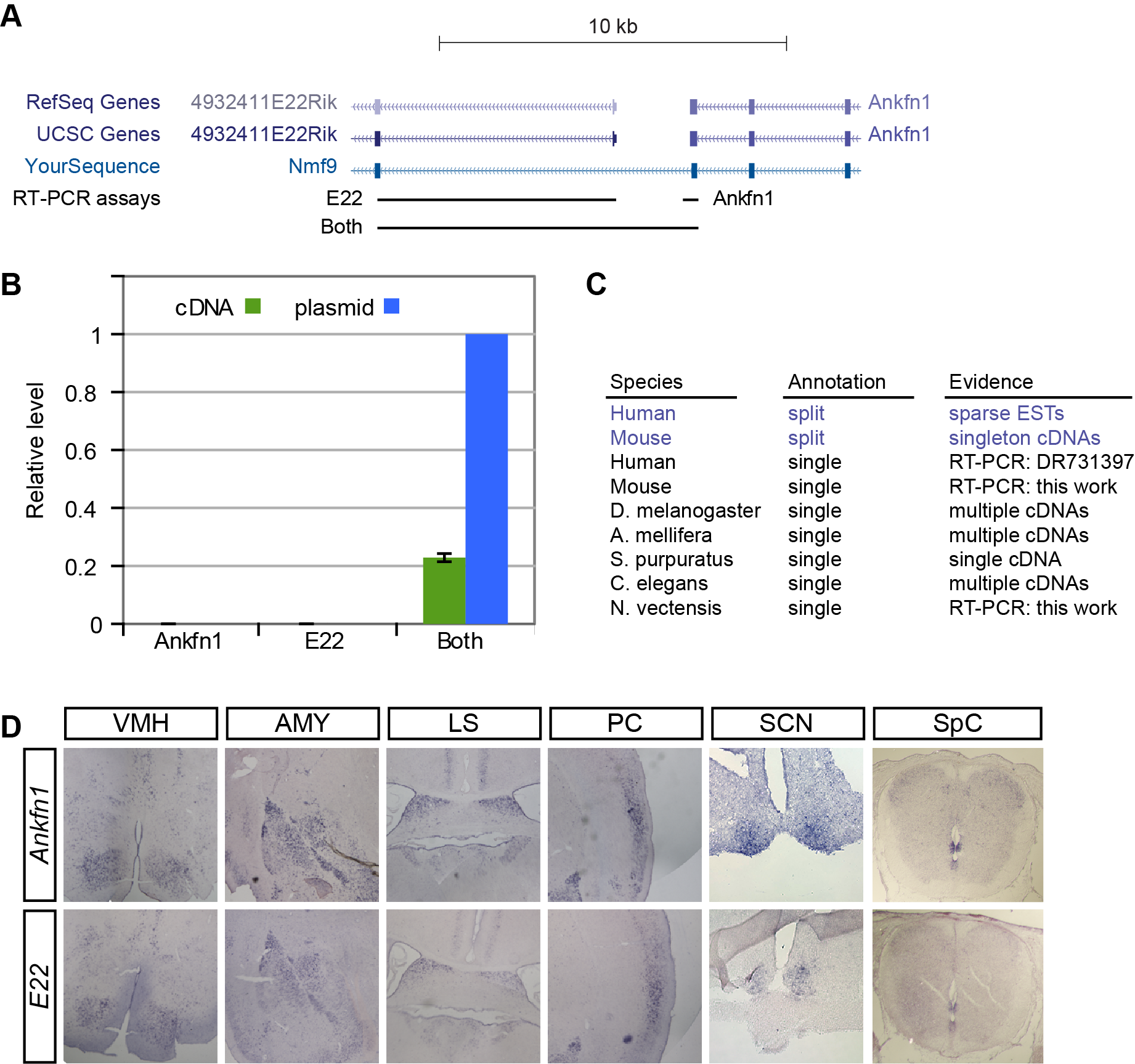

Supplement: S2 Fig — (A) UCSC Genome Browser shows minus-strand transcripts for the 3’ exons of Ankfn1 and 5’ exons of E22 compared to BLAT alignment of empirically determined Nmf9 mRNA sequence. Positions of primers used for RT-PCR are indicated. (B) Relative quantities for real-time fluorescence measurements (SYBR) of RT-PCR products from the indicated assays from either brain cDNA or cloned plasmid. (C) Publicly deposited cDNA sequences for insect (D. melanogaster, A. mellifera), echinoderm (S. purpuratus), nematode (C. elegans) and cnidarian (N. vectensis) also support a single transcript across segments homologous to Ankfn1 and E22. (D) In situ hybridization patterns were indistinguishable for Ankfn1 and E22 probes in ventromedial hypothalamus (VMH), amygdala (AMY), lateral septum (LS), piriform cortex (PC), suprachiasmatic nuclei (SCN) and spinal cord (SpC). This mirrors data for both annotations in the Allen Brain Atlas (Fig 3B). (TIF) [file pgen.1005344.s008.tif]

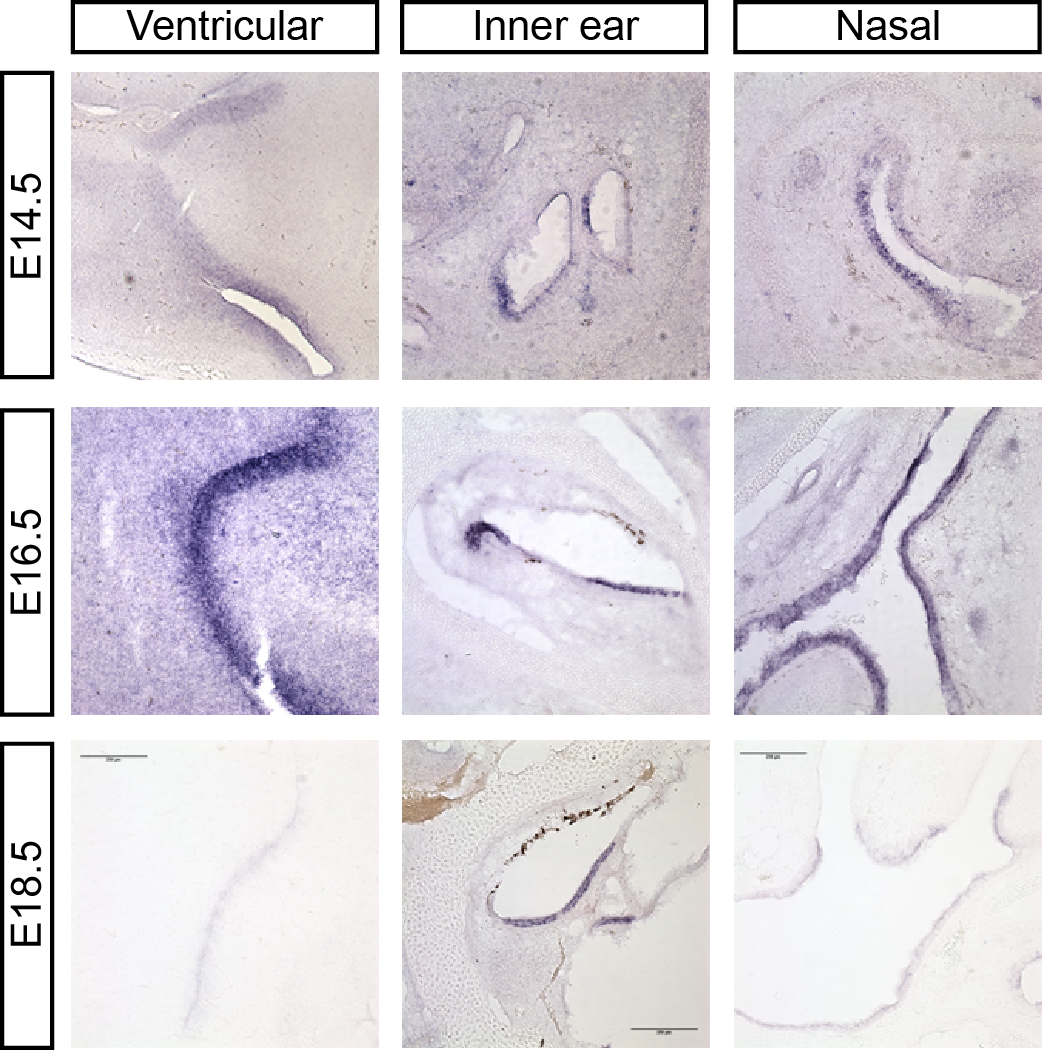

Supplement: S3 Fig — Nmf9 RNA was detected by in situ hybridization as early as E14.5 in ventricular zones, inner ear, and nasal epithelium. (TIF) [file pgen.1005344.s009.tif]

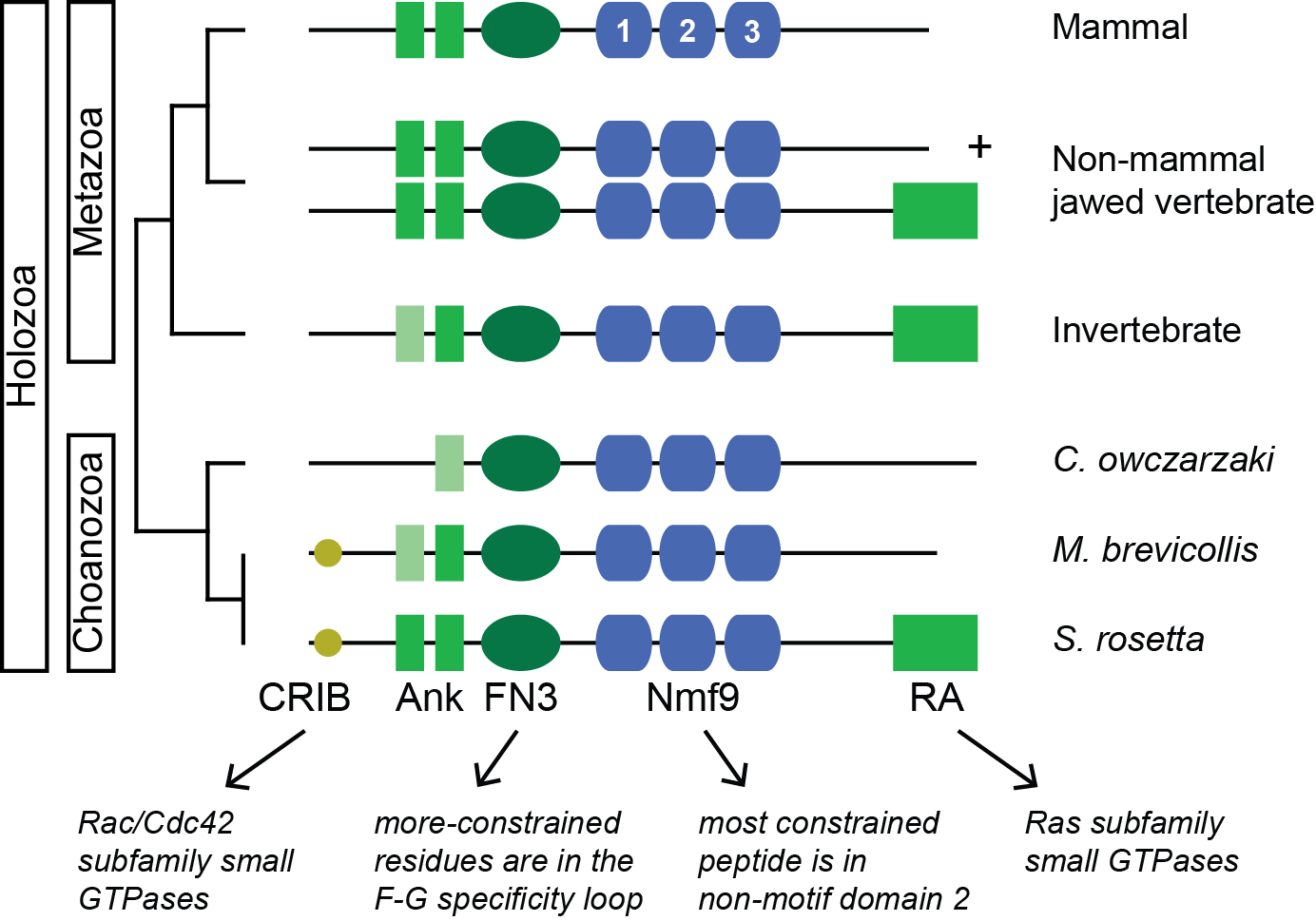

Supplement: S4 Fig — Known motifs are indicated in green. Highly consereved domains novel to the Nmf9 homology group are shown in blue. An aminoterminal CRIB domain was found only in the two choanoflagellae species, while inclusion or loss of a ras-association domain (RA) appears not to be monophyletic. Vertebrate species basal to mammals had paralogous copies, one with and one without the RA domain in each species. All mammals examined had only one copy, which never had an identifiable RA domain. Conservation of the first ANK repeat, and consensus match to the motif definition were poor or absent in some invertebrate and choanozoa species. (TIF) [file pgen.1005344.s010.tif]

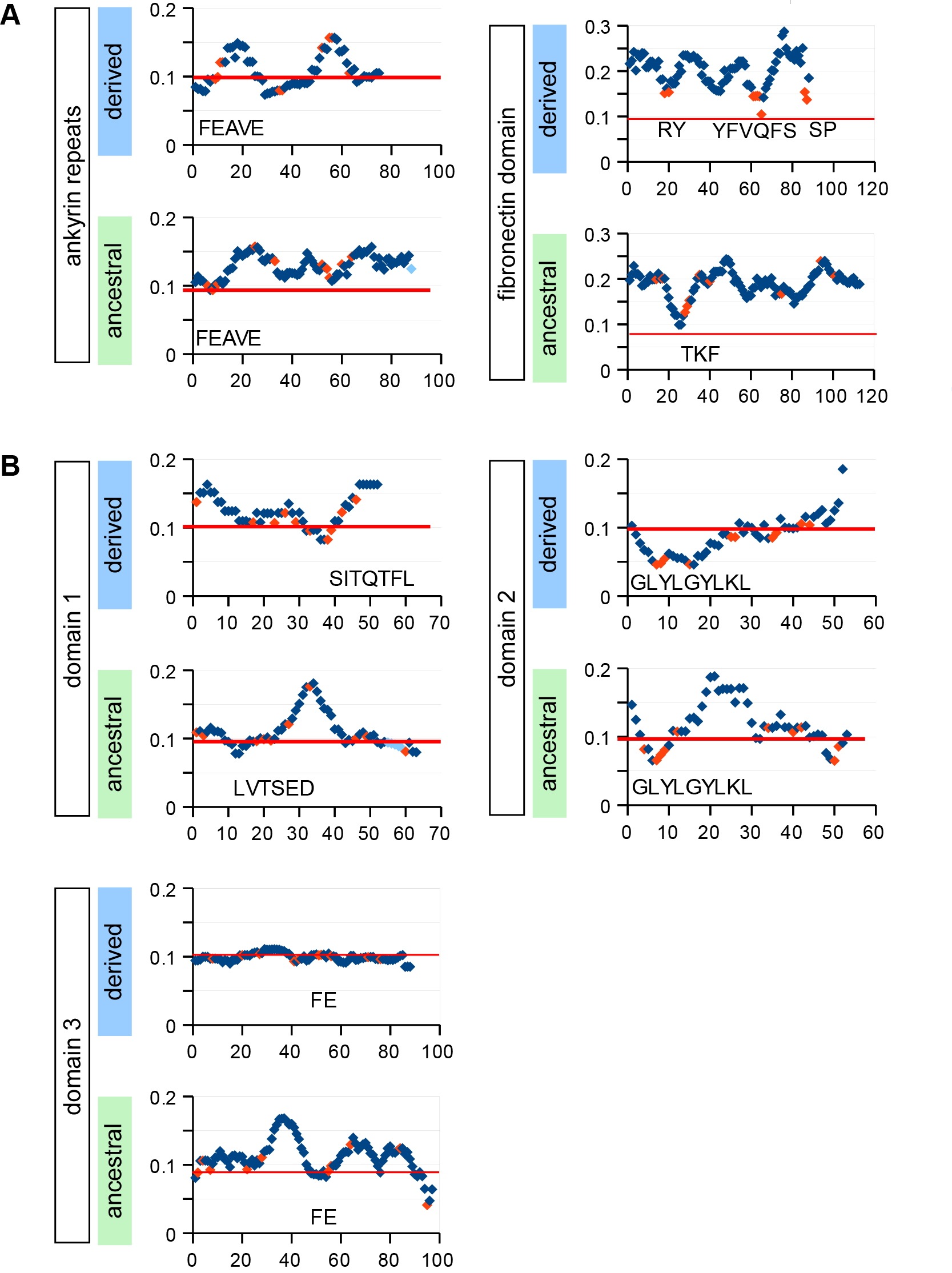

Supplement: S5 Fig — High-resolution sliding window analysis of domains, with inclusion of species that lacked full-length sequence. Separate analyses were performed for ancestral homologs and vertebrate-derived homologs. Y-axis shows dN/dS values calculated using HyPhy [56]. The 10 residues with smallest dN in each analysis are plotted in orange. Light blue points indicate gaps in sequence. Amino acid residues are given for regions of highest conservation. (A) Annotated ankyrin and fibronection type III motifs are plotted. For the ankyrin repeats, N = 53 species for derived homologs, 47 for ancestral homologs. For fibronectin type III repeats, N = 50 derived, 50 ancestral homologs. (B) The three highly conserved domains that do not align with known motif definitions are plotted. For domain 1, N = 60 species for derived homologs, 52 for ancestral. For domain 2, N = 54 derived, 49 ancestral. For domain 3, 49 derived, 48 ancestral. (TIF) [file pgen.1005344.s011.tif]

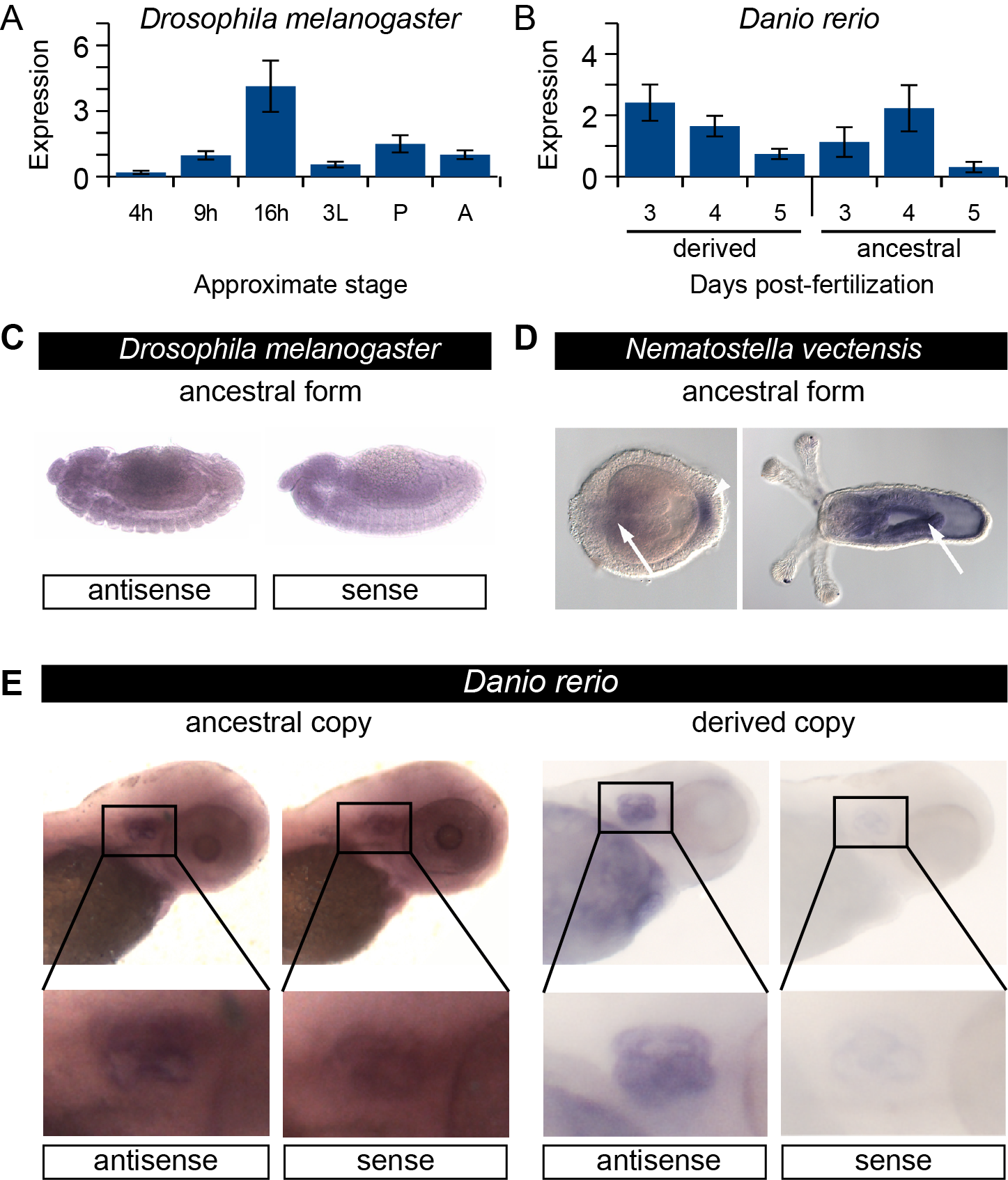

Supplement: S6 Fig — Nmf9 homologues were expressed during development in (A) fruit fly and (B) zebrafish. Samples included 3 biological replicates for fruit fly, but only technical replicates of single samples for each zebrafish stage and paralogous gene. (C) In situ hybridization to Drosophila embryos showed broad CG45058 expression, including CNS, agreeing with stage-specific expression in the BDGP in situ database (http://insitu.fruitfly.org/). (D) Expression in sea anemone N. vectensis planulae (left) includes endoderm (arrow) and some apical tuft cells. In polyps, expression appears uniform throughout the endoderm (arrow) and mesenteries. (E) In zebrafish embryos, ancestral paralog retained a broad but weak expression pattern that includes expression in the developing inner ear (box and inset). The derived paralog showed a cleaner, more restricted pattern. (TIF) [file pgen.1005344.s012.tif]

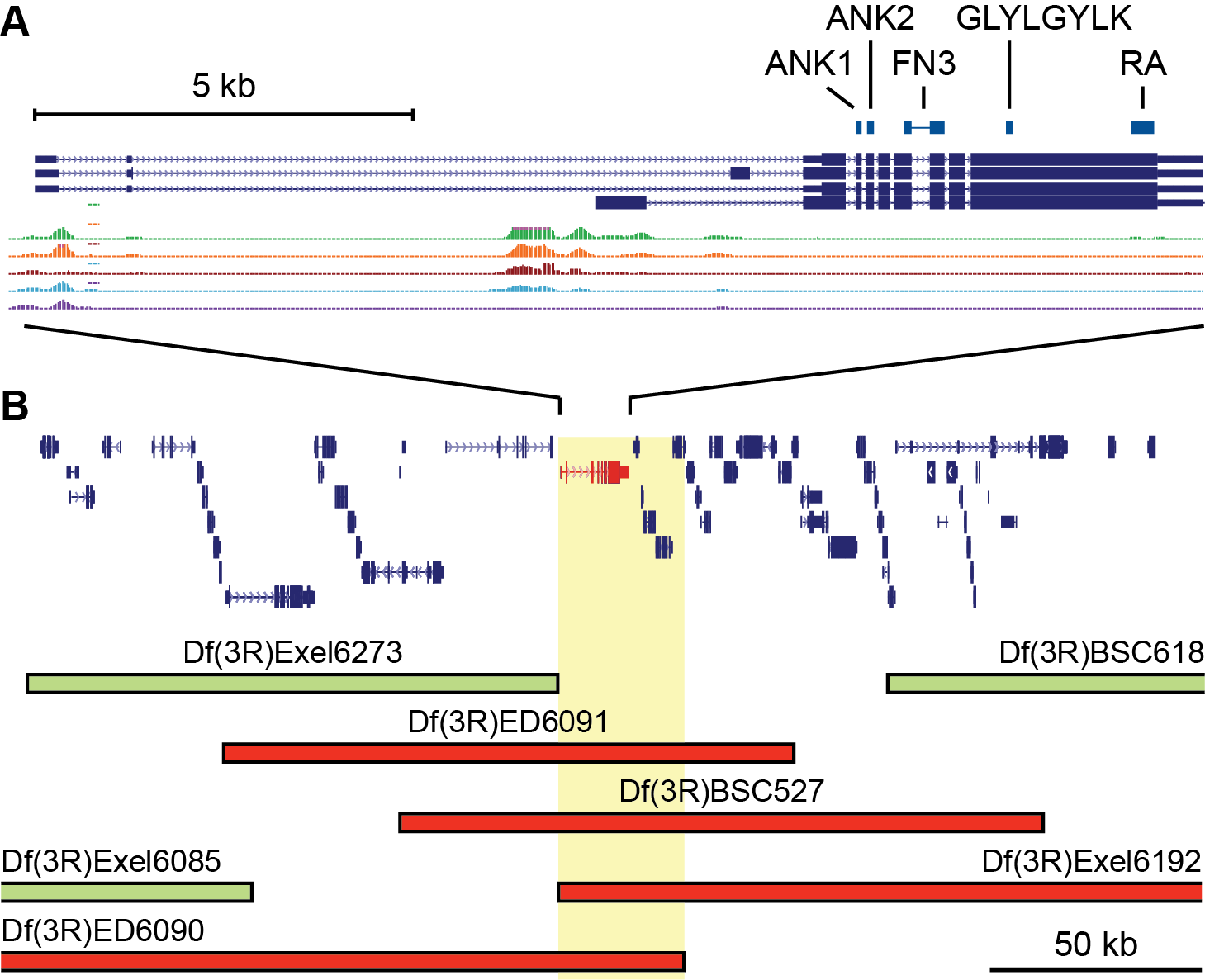

Supplement: S7 Fig — (A) Schematic of the CG45058 locus shows exon position of encoded protein domains (blue) across transcripts (navy) from the UCSC browser. Chromatin accesibility to DNase I at stage 5 (green), 9 (orange), 10 (red), 11(blue) and 14 (purple) embryos suggests dynamic regulation of alternative transcripts. (B) UCSC transcripts (navy) in the interval around CG45058 (red) on Drosophila chromosome 3R are shown relative to deficiencies that complement (green bars) or fail to complement (red bars) frameshift mutation at conserved domain 2 (GLYLGYLK). The inferred interval (yellow box) defined by the shared breakpoint of Df(3R)Exel6273 and Df(3R)Exel6192 and the right breakpoint of Df(3R)ED6090 contains parts of 6 transcription units. (TIF) [file pgen.1005344.s013.tif]
